# F0F1 ATP synthase regulates extracellular calcium influx in human neutrophils by interacting with Ca_v_2.3 and modulates neutrophil accumulation in the lipopolysaccharide-challenged lung

**DOI:** 10.1186/s12964-020-0515-3

**Published:** 2020-02-04

**Authors:** Baoyi Zhu, Zhengfu Feng, Yan Guo, Tian Zhang, Ai Mai, Zhanfang Kang, Ting Weijen, Dai Wang, Dazhong Yin, Dongxing Zhu, Jun Gao

**Affiliations:** 1 Department of Basic Medical Research, The Sixth Affiliated Hospital of Guangzhou Medical University, Qingyuan, 511518 Guangdong China; 2 Clinical Laboratory of Dongcheng People’s Hospital, Dong guan, 523007 Guangdong China; 3 Graduate Institute of Basic Medical Science, China Medical University, Taichung, 40402 Taiwan; 4 State Key Laboratory of Molecular Vaccinology and Molecular Diagnostics of Xiamen University, Xiamen, 361102 Fujian China; 5 Key Laboratory of Cardiovascular Diseases, School of Basic Medical Sciences, Guangzhou Medical University, Guangzhou, 511436 Guangdong China

**Keywords:** F0F1 ATP synthase, Neutrophil, Voltage-gated calcium channel, Inflammation, Lung

## Abstract

**Background:**

Neutrophils form the first line of innate host defense against invading microorganisms. We previously showed that F0F1 ATP synthase (F-ATPase), which is widely known as mitochondrial respiratory chain complex V, is expressed in the plasma membrane of human neutrophils and is involved in regulating cell migration. Whether F-ATPase performs cellular functions through other pathways remains unknown.

**Methods:**

Blue native polyacrylamide gel electrophoresis followed by nano-ESI-LC MS/MS identification and bioinformatic analysis were used to identify protein complexes containing F-ATPase. Then, the identified protein complexes containing F-ATPase were verified by immunoblotting, immunofluorescence colocalization, immunoprecipitation, real-time RT-PCR and agarose gel electrophoresis. Immunoblotting, flow cytometry and a LPS-induced mouse lung injury model were used to assess the effects of the F-ATPase-containing protein complex in vitro and in vivo.

**Results:**

We found that the voltage-gated calcium channel (VGCC) α2δ-1 subunit is a binding partner of cell surface F-ATPase in human neutrophils. Further investigation found that the physical connection between the two proteins may exist between the F1 part (α and β subunits) of F-ATPase and the α2 part of VGCC α2δ-1. Real-time RT-PCR and PCR analyses showed that Ca_v_2.3 (R-type) is the primary type of VGCC expressed in human neutrophils. Research on the F-ATPase/Ca_v_2.3 functional complex indicated that it can regulate extracellular Ca^2+^ influx, thereby modulating ERK1/2 phosphorylation and reactive oxygen species production, which are typical features of neutrophil activation. In addition, the inhibition of F-ATPase can reduce neutrophil accumulation in the lungs of mice that were intratracheally instilled with lipopolysaccharide, suggesting that the inhibition of F-ATPase may prevent neutrophilic inflammation-induced tissue damage.

**Conclusions:**

In this study, we identified a mechanism by which neutrophil activity is modulated, with simultaneous regulation of neutrophil-mediated pulmonary damage. These results show that surface F-ATPase of neutrophils is a potential innate immune therapeutic target.

**Graphical abstract:**

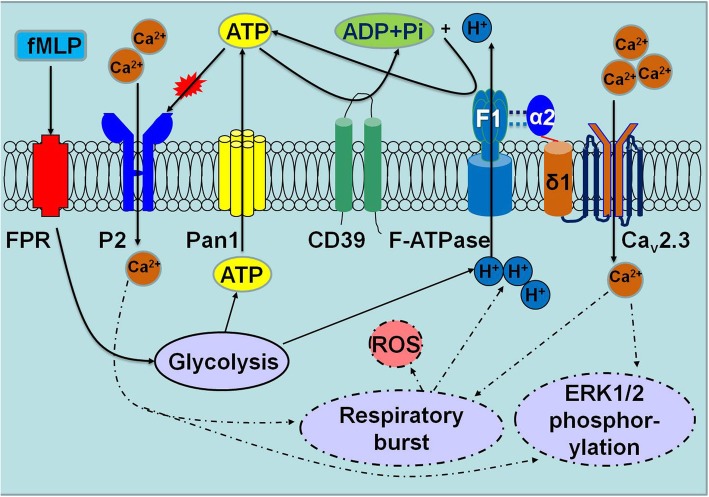

## Background

Human neutrophils possess a tubular mitochondrial network, which is different from the well-known granular mitochondria [1]. Studies on mitochondrial function in neutrophils commonly assumed that this mitochondrial system does not participate in oxidative phosphorylation. Because the energy requirements of neutrophils are mostly provided by anaerobic glycolysis and because mitochondria lose almost all cytochrome C during neutrophil maturation, ATP cannot be effectively produced by oxidative phosphorylation [2, 3]. In addition, obtaining energy from anaerobic respiration is more appropriate for neutrophil function because inflamed or infected sites are often in an anoxic state [4]. However, it has been reported that treatment of neutrophils with oligomycin, an F0F1 ATP synthase (F-ATPase)-specific inhibitor, affects cell chemotaxis, cell morphology, neutrophil extracellular trap formation, cell activation, etc. [1, 5, 6]. F-ATPase is commonly assumed to be respiratory chain complex V in the inner membrane of mitochondria; this complex consists of a transmembrane proton transport domain, F0, and an ATP synthesis catalytic domain, F1 [7]. The key role of F-ATPase is transferring protons from the intermembrane space to the matrix for ATP synthesis in mitochondria [8]. Theoretically, the mitochondrial membrane potential will increase in the presence of oligomycin because oligomycin can hyperpolarize mitochondria by blocking proton re-entry from the intermembrane space to the matrix via its effects on F0-ATP synthase [9]; however, Fossati et al. found that oligomycin had no effect on the mitochondrial membrane potential in neutrophils [1]. Therefore, strictly speaking, F-ATPase rather than mitochondria participates in the regulatory process described above.

Proteomic studies have reported the presence of F-ATPase in plasma membranes purified from human neutrophils [10, 11]; however, due to the limitations of the protein database, the mass spectrometry (MS) results still identify F-ATPase as a mitochondrial protein, although it has been reported that F-ATPase is expressed on the surface of different cells [12–17]. Previous studies have reported that F-ATPase is a potential cell surface receptor for angiostatin, which can regulate neutrophil migration [18]; however, the detailed mechanism remains unclear. Our previous studies found F-ATPase in the plasma membrane of red blood cells, which do not contain mitochondria [19]. Thus, it is tempting to hypothesize that F-ATPase can be expressed in the plasma membrane of neutrophils because both red blood cells and neutrophils are differentiated from hematopoietic stem cells. Further research by our group confirmed that F-ATPase can be expressed not only in mitochondria but also on the surface of neutrophils and that this protein acts as an intracellular pH regulator and extracellular ATP generator, thereby modulating cell adhesion and migration [20]. Whether F-ATPase performs cellular functions through other pathways remains unknown.

In this study, a neutrophil plasma membrane protein that interacts with F-ATPase was identified, and a novel pathway by which F-ATPase regulates neutrophil Ca^2+^ influx was elucidated, increasing our understanding of the role of F-ATPase in neutrophil activation. In addition, we further investigated the role of F-ATPase in the accumulation of neutrophils in lipopolysaccharide (LPS)-induced pulmonary damage in mouse models and observed that the inhibition of F-ATPase can reduce neutrophil accumulation and neutrophilic inflammation. These results provide new insights into the treatment of neutrophil-related diseases.

## Methods

### Chemicals

Sodium dodecyl sulfate (SDS), bovine serum albumin (BSA) and phenylmethane sulfonyl fluoride (PMSF) were purchased from Amresco (Solon, OH, USA). Phosphate-buffered saline (PBS), RIPA Lysis Buffer, SDS-PAGE kit and SDS loading buffer were obtained from Beyotime (Shanghai, China). Oligomycin A was purchased from Selleckchem (Houston, TX, USA). SNX482 was obtained from Alomone Labs (Jerusalem, Israel). Agarose, ethylenediaminetetraacetic acid (EDTA), methanol, paraformaldehyde, Tween 20, thenoyltrifluoroacetone (TTFA), polyformaldehyde, phosphatase inhibitor cocktail 3, dimethyl sulfoxide (DMSO), 4′,6-diamidino-2-phenylindole (DAPI) and N-formyl-Met-Leu-Phe (fMLP) were purchased from Sigma-Aldrich (St. Louis, MO, USA). Fluo-4/AM, Ca^2+^-free HBSS, Ca^2+^-containing HBSS, digitonin and Coomassie blue G-250 were obtained from Invitrogen (Carlsbad, CA, USA). Unless otherwise stated, all concentrations shown are the final concentrations.

### Isolation of neutrophils

The Institutional Review Board of the Sixth Affiliated Hospital of Guangzhou Medical University approved this work. Neutrophils were isolated from heparinized blood of healthy donors aged 25–35 years by using the Human Peripheral Blood Neutrophil Isolation Kit (Beyotime, Shanghai, China) according to the manufacturer’s instructions. During isolation of the cells, pyrogen contamination and excessive mechanical stimulation were carefully avoided. The average purity of neutrophils was > 95%, as determined by Wright-Giemsa staining, and the average viability of isolated cells was > 98%, as assessed by trypan blue dye exclusion. After isolation, the cells were resuspended in Ca^2+^-free HBSS at 5 × 10^6^ cells/ml and stored at room temperature until use (< 1 h).

### BN-PAGE and immunoblot analysis

Antibodies against F-ATPase that can be used for immunoprecipitation are unavailable; thus, F-ATPase binding proteins were investigated by blue native polyacrylamide gel electrophoresis (BN-PAGE) according to the instructions of the native page sample prep kit (Invitrogen, Carlsbad, CA, USA) and procedures described previously [21]. Briefly, the plasma membranes of neutrophils were prepared as previously described [22] and were subsequently lysed in NativePAGE Sample Buffer (Invitrogen, Carlsbad, CA, USA) containing 2% digitonin, incubated on ice for 1 h and then centrifuged at 20,000 g for 30 min at 4 °C. Loading buffer with Coomassie blue G-250 was added to the supernatant to make the protein negatively charged. Samples (80 μg) were loaded into each well of NativePAGE Novex 4–16% Bis-Tris gels (Invitrogen, Carlsbad, CA, USA). Electrophoresis was performed at 4 °C with a constant voltage of 150 V for 120 min. The dark blue cathode buffer was exchanged with light blue cathode buffer after 30 min of electrophoresis. Each lane of the BN-PAGE gel was carefully cut out, and the excised bands were stored in sealed tubes at 4 °C after electrophoresis. Western blotting was used to identify the position of the F-ATPase-containing protein complex in the lane from the BN-PAGE gel. Proteins in one excised lane were transferred to a PVDF membrane (Millipore, Bedford, MA, USA), which was blocked with 10% skim milk in TBST. F-ATPase was detected with a mouse monoclonal antibody against the F-ATPase F1 β-subunit (1:2000, 612,519, BD Biosciences, CA, USA) and an HRP-conjugated rabbit anti-mouse IgG H & L secondary antibody (1:2000, ab6728, Abcam, Cambridge, UK). The signal was collected using an ECL kit (Millipore, Bedford, MA, USA) via a DNR chemiluminescence imaging system.

### Nano-HPLC MS/MS analysis

The bands of interest from three lanes in gels that were run in parallel with those used for western blot analysis were excised according to the position of the F-ATPase-containing protein complex observed in the western blot analysis described above. The in-gel digestion of the excised band was performed as described previously [19]. Briefly, the bands were destained in 25 mM NH_4_HCO_3_ (Sigma-Aldrich, St. Louis, MO, USA) with 50% acetonitrile (ACN) (Sigma-Aldrich, St. Louis, MO, USA). Then, the bands were washed with ddH_2_O 4 times. After washing with 50 and 100% ACN in 25 mM NH_4_HCO_3_ sequentially, the bands were dried in a Speed Vac and rehydrated in 10 μl of 25 mM NH_4_HCO_3_ with 0.01 μg/μl trypsin (Promega, Madison, WI, USA). After incubation at 37 °C for 16 h, the peptides were extracted twice with 5% formic acid (Sigma-Aldrich, St. Louis, MO, USA) and a 66.7% ACN solution via 20 min of sonication and then concentrated in the Speed Vac.

Reversed-phase nano-LC-MS/MS analysis was performed on the Eksigent nanoLC-Ultra™ 2D System (AB SCIEX, Concord, ON, Canada). The lyophilized fractions were suspended in 5% ACN and 0.1% formic acid and loaded onto a ChromXP C18 (3 μm, 120 Å) nanoLC trap column. The online trapping and desalting procedure was carried out at 2 μl/min for 10 min with 100% solvent A. The solvents were composed of water/ACN/formic acid (A, 95/5/0.1%; B, 5/95/0.1%). Then, an elution gradient of 5–40% ACN (0.1% formic acid) in 25 min was employed on an analytical column (75 μm 15 cm, C18, 3 μm, 120 Å, ChromXP Eksigent). LC MS/MS analysis was performed on a Triple TOF 5600 System (AB SCIEX, Concord, ON, Canada) fitted with a Nanospray III source (AB SCIEX, Concord, ON, Canada). Data were acquired using an ion spray voltage of 2.4 kV, a curtain gas of 30 psi, a nebulizer gas of 5 psi, and an interface heater temperature of 150 °C. The MS was performed with time-of-flight (TOF)-MS scans with a range from 400 to 1250 m/z. For information-dependent acquisition, survey scans started from 100 to 1500 m/z and were acquired in 250 ms, and as many as 30 product ion scans (60 ms) were collected if the product ion exceeded the threshold of 200 counts per second (counts/s) and with a + 2 to + 5 charge state. A rolling collision energy setting was applied to all precursor ions for collision-induced dissociation. Dynamic exclusion was set for ½ of peak width (approximately 16 s).

### Data analysis and bioinformatics

The raw data files of MS/MS data were processed and analyzed using PEAKS Studio 8.5 software (version 8.5, Bioinformatics Solutions Inc., Waterloo, Canada). Further information on the identified proteins was in accordance with the UniProt database (http://www.uniprot.org). The search parameters were set as follows: Database, *Homo sapiens* downloaded from UniProt (20,211 proteins in total after redundancy removal); Enzyme, trypsin, allowing up to one missed cleavage. The peptide mass tolerance was 20 ppm, and the MS/MS mass tolerance was 0.1 Da; the variable modification parameter was oxidation (Met). We basically selected the candidate peptides that conformed to the filtering criteria, with a false discovery rate (FDR) of peptides less than 5%. Proteins that were identified with at least one “unique” peptide showing a -10lgP value higher than 20 were accepted without any manual validation.

### One-dimensional (1D) and two-dimensional (2D) immunoblotting analysis and immunofluorescence colocalization analysis

Protein complexes in one excised lane of BN-PAGE (1D) were transferred to a PVDF membrane, which was then blocked in 10% skim milk in TBST. Voltage-gated calcium channel (VGCC) α2δ-1 was detected with a rabbit polyclonal antibody against the VGCC α2δ-1 subunit (1:200, C5105, Sigma-Aldrich, St. Louis, MO, USA) and an HRP-conjugated goat anti-rabbit IgG H & L (1:2000, ab6721, Abcam, Cambridge, UK). The signal was collected using an ECL kit (Millipore, Bedford, MA, USA) via a DNR chemiluminescence imaging system. For 2D immunoblot analysis, one excised lane of the BN-PAGE gel was equilibrated for 30–60 min in SDS loading buffer at room temperature. Then, the equilibrated lane was positioned horizontally on the top surface of the 10% SDS running gel and fixed with 1% agarose. Proteins were transferred to a PVDF membrane after the run was over, and the membrane was blocked in 10% skim milk in TBST. Then, the membrane was incubated with the antibody against the VGCC α2δ-1 subunit (1:200, Sigma-Aldrich, St. Louis, MO, USA) and an HRP-conjugated goat anti-rabbit IgG H & L (1:2000, Abcam, Cambridge, UK). After the signal was detected, the same PVDF membrane labeled with the α2δ-1 subunit antibody was washed in TBST. Then, the membrane was incubated with an antibody against the F-ATPase F1 β-subunit (1:2000, 612,519, BD Biosciences, CA, USA) and an HRP-conjugated rabbit anti-mouse IgG H & L secondary antibody (1:2000, Abcam, Cambridge, UK). The signal was collected as described above.

To observe the distribution of cell surface ATP synthase and VGCC α2/δ-1, neutrophils were allowed to adhere to 20 μg/ml fibronectin-coated coverslips for 15 min. Suspended cells were removed by several gentle washes with PBS. The adherent cells were fixed in 4% paraformaldehyde for 30 min without permeabilization in Triton X-100. The neutrophils were then washed with PBS and blocked in 5% BSA-containing PBS for 1 h, followed by incubation at 4 °C overnight with a mouse monoclonal F-ATPase F1 β-subunit antibody diluted in PBST (1:100, 612,519, BD Biosciences, CA, USA). Then, the cells were washed and incubated for 1 h in the dark at 37 °C with a goat anti-mouse IgG Alexa Fluor 546 antibody (1:500, 1,736,960, Molecular Probes/Invitrogen, Carlsbad, CA, USA). In addition, the cells were washed and incubated with a rabbit polyclonal antibody against the VGCC α2δ-1 subunit (1:50, Sigma-Aldrich, St. Louis, MO, USA) diluted in PBST at 4 °C overnight, followed by several washes and incubation for 1 h in the dark at 37 °C with a goat anti-rabbit IgG Alexa Fluor 488 antibody (1:500, 1,853,312, Molecular Probes/Invitrogen, Carlsbad, CA, USA) in PBST. The stained neutrophils were then labeled with 1 μg/ml DAPI for 3 min at room temperature. The labeled cells were washed several times with PBST to reduce the background and photographed under a confocal microscope (SP8, Leica, Mannheim, Germany).

### Immunoprecipitation and nano-HPLC MS/MS analysis of proteins

To further confirm whether there is a physical connection between F-ATPase and the VGCC α2δ-1 subunit, immunoprecipitation analysis was performed. The plasma membrane of neutriphils was lysed in NativePAGE Sample Buffer (Invitrogen, Carlsbad, CA, USA) containing 2% digitonin. Immunoprecipitation was performed according to the instructions of the IP kit (Thermo Scientific). Briefly, the plasma membrane protein lysate was combined with 10 μg of antibody against the α2 part of the VGCC α2δ-1 (Mouse monoclonal-CACNA2D1 antibody, ab2864, Abcam), and the reaction volume was adjusted to 500 μL with the NativePAGE Sample Buffer; mouse IgG (ab18413, Abcam) was used as a control. Then, we incubated the reaction overnight at 4 °C. Then, 0.25 mg of prewashed Pierce™ protein A/G magnetic beads (88,802, Thermo Scientific) was added to the proteins/antibody mixture and incubated at room temperature for 1 h. Then, the beads were collected with a magnetic stand and washed three times with wash buffer and divided into two parts. One part was stored at − 80 °C until further use for nano-HPLC MS/MS analysis, and the left part was saved for immunoblot analysis. For nano-HPLC MS/MS analysis, we followed previously described procedures [23] with few modifications. Briefly, protein-bound magnetic beads were mixed with 200 μl of 8 M urea in a Nanosep Centrifugal Device. The device was centrifuged at 14,000 g for 20 min at room temperature. All following centrifugation steps were performed under the same conditions. Then, the concentrate was diluted with 200 μl of 8 M urea in 0.1 M Tris-HCl, pH 8.5, and the device was centrifuged again. After this, 100 μl of 0.05 M iodoacetamide in 8 M urea and 0.1 M Tris-HCl, pH 8.5 were added to the concentrate, followed by centrifugation. The resulting concentrate was diluted with 200 μl of 8 M urea in 0.1 M Tris-HCl, pH 8.0 and concentrated again. Then, the concentrate was subjected to proteolytic digestion with 0.01 μg/μl trypsin solution. The digests were collected by centrifugation, and the filter device was rinsed with 50 ml 0.5 M NaCl and centrifuged. The subsequent nano-HPLC MS/MS and bioinformatics analysis was the same as mentioned above. For immunoblot analysis, protein-bound magnetic beads were boiled in SDS-page reducing sample buffer. The supernatant was saved after magnetically separating the beads. Mouse monoclonal antibodies against the F-ATPase F1 α-subunit/β-subunit (1:2000, 612,516/612519, BD Biosciences, CA, USA) were used as primary antibodies, and an IPKine™ HRP-conjugated goat anti-mouse IgG light chain specifically bound antibody (1:5000, Abbkine, GZ, China) was used as secondary antibody. The signal was collected as described above.

### RNA isolation, quantitative real-time RT-PCR and agarose gel electrophoresis

The relative mRNA expression levels of all known kinds of mammalian VGCC were determined in human neutrophils by real-time RT-PCR. Total neutrophil RNA was extracted using RNAiso Plus (TaKaRa, Dalian, China) according to the manufacturer’s instructions. For real-time quantification, first strand cDNA was prepared using the PrimeScriptTM RT Master Mix Kit (TaKaRa, Dalian, China). Real-time PCR was performed using the SYBR Green Kit (TaKaRa, Dalian, China) on a CFX Connect™ Real-Time System (Bio-Rad, Hercules, CA, USA). For the agarose gel electrophoresis assay, the amplicons of quantitative real-time RT-PCR without melt curve analysis were loaded into an agarose gel after mixing with SYBR safe (Invitrogen, Carlsbad, CA, USA). After running, the gel was imaged by a gel imaging system (Quantum, Vilber Lourmat, Marne La Vallee, France). All the data were normalized to the expression levels of GAPDH in each sample. The primers obtained from PrimerBank (https://pga.mgh.harvard.edu/primerbank) are listed in Additional file 1.

### Calcium influx analysis

Fresh isolated neutrophils were incubated in Ca^2+^-free HBSS and a 3 μM concentration of the fluorescent Ca^2+^ indicator Fluo-4/AM for 30 min at 37 °C. The labeled cells were washed with Ca^2+^-free HBSS, and the dye was allowed to de-esterify for another 20 min at room temperature. Then, the labeled cells were collected and resuspended in Ca^2+^-containing HBSS and stimulated as indicated in the text (1 × 10^6^ cells/ml). Fluo-4-loaded cells in Ca^2+^-free HBSS were used as a negative control for Ca^2+^ influx. After pre-warming for 5 min at 37 °C, the relative fluorescence intensity (RFI) of labeled cells was measured with a fluorescence spectrometer (LS55, PerkinElmer, Waltham, MA, USA) at an excitation of 488 nm and emission of 525 nm followed by activation by 100 nM fMLP.

### Erk1/2 phosphorylation analysis

Fresh isolated neutrophils (1 × 10^6^ cells/ml) were resuspended in Ca^2+^-containing HBSS and stimulated with drugs as indicated in the text for 30 min at room temperature. Then, the cells were activated with 100 nM fMLP for 1 min after pre-warming for 5 min at 37 °C, followed by the addition of a 5-fold volume of ice-cold HBSS and storage on ice to stop the reaction. The cells in Ca^2+^-free HBSS were used as negative controls for Ca^2+^ influx. For Erk1/2 phosphorylation analysis, neutrophils were lysed in RIPA with phosphatase inhibitor cocktail 3 and PMSF. Equal amounts of proteins from lysed cells or were quantified and then subjected to SDS-PAGE (4.8% stacking gel and 10% resolving gel). The next western blot operation is similar to the one mentioned above except rabbit monoclonal antibodies against phospho-Erk1/2 (1:1000, 4370S, CST, Danvers, MA, USA) and Erk1/2 (1:1000, 4695S, CST, Danvers, MA, USA) were used as the primary antibodies.

### Reactive oxygen species (ROS) formation

The ROS fluorescent indicator dihydrorhodamine 123 (DHR123, Molecular Probes) was used to monitor ROS levels during cell activation, and the procedure was modified as described [24]. Briefly, freshly isolated neutrophils (1 × 10^6^ cells/ml) were incubated in 1 μM DHR123 in Ca^2+^-free HBSS at 37 °C for 20 min. After washing with Ca^2+^-free HBSS, the DHR123-loaded cells were resuspended in Ca^2+^-containing HBSS and treated with 100 nM fMLP for 15 min at 37 °C. To stop the reaction, a 5-fold volume of ice-cold Ca^2+^-containing HBSS was added and kept at 4 °C in the dark. The aggregated cells were removed by filtration through a 35-μm cell strainer snap cap on a flow cytometry tube (Falcon, Corning, Cambridge, MA). Then, neutrophils were recognized via a BD Accuri C6 Flow Cytometer (BD Biosciences, San Jose, CA, USA) on the basis of forward light scatter (FSC) and side light scatter (SSC), which identified neutrophils and excluded other cell types (a few contaminating lymphocytes and red blood cells), dead cells and debris from the analysis. Appropriate gates were set to take the changes in the light-scattering pattern of fMLP-activated neutrophils into consideration during analysis. FL1 channels (green fluorescence) were chosen to detect the fluorescence of rhodamine 123, which was oxidized from DHR123 by ROS.

### LPS-induced acute lung injury mouse model

The Institutional Review Board of the Sixth Affiliated Hospital of Guangzhou Medical University approved the protocols for this study. Mice (Kunming, 10 weeks old) were purchased from The Southern Medical University Experimental Animal Center. The acute lung injury mouse model was modified as previously described [25]. Briefly, the mice were anesthetized by the inhalation of oxygen with 3% isoflurane in a closed anesthesia box. Subsequently, the mice were immobilized on an animal operating table with the mask placed near one side of the nose to facilitate intubation from the mouth, with the isoflurane concentration adjusted to 5% to maintain anesthesia. The incisors were attached with a rubber band. Then, the mouth was opened, and the tongue was pulled out, followed by the insertion of a sterile 6# gavage needle into the trachea through the glottis and LPS instillation (LPS from *E. coli* O111:B4, 5 mg/kg body weight, Sigma-Aldrich, St. Louis, MO, USA) followed by 50 μl of air. Then, the mice were suspended by their front legs to help instillate the LPS deep into the lungs before being placed back into the cage for recovery. For the F-ATPase inhibition treatment, oligomycin A was injected intraperitoneally (500 μg/kg body weight) at 0 and 12 h after the instillation of LPS, with PBS infused into the lungs as a control.

### Neutrophil accumulation analysis

Mice were anesthetized and instilled with LPS as described above and were euthanized by CO_2_ after 24 h. For bronchoalveolar lavage (BAL), after carefully exposing the trachea, a 6# gavage needle was inserted into the trachea from the glottis to avoid blood flow into the trachea due to tracheotomy. Subsequently, the trachea and the inserted gavage needle were tightened together. Bronchoalveolar lavage (BAL) was performed with HBSS supplemented with 1 mM EDTA (1 ml × 5) for each group. The BAL fluid (BALF) was centrifuged at 400 g for 10 min, and the total number of cells in the BALF was determined by hemocytometry. The percentage of neutrophils was determined using Wright-Giemsa stain with cytospin preparations. In addition, emigrated neutrophils in the alveolar air spaces were analyzed by morphometric analysis of tissue sections. The left lungs were fixed in 10% (v/v) neutralized and buffered formaldehyde and embedded in paraffin, after which 4-μm thick sections were cut and stained with hematoxylin and eosin (H&E).

### Statistical analyses

In the present study, all data are shown as the mean ± S.D. of three similar experiments. The data were analyzed using one-way ANOVA with Tukey’s multiple comparison test with SPSS 19.0 software. Differences were considered significant at *P* < 0.05.

## Results

### The VGCC α2δ-1 subunit was identified as a binding partner of cell surface F-ATPase

**Fig. 1:**
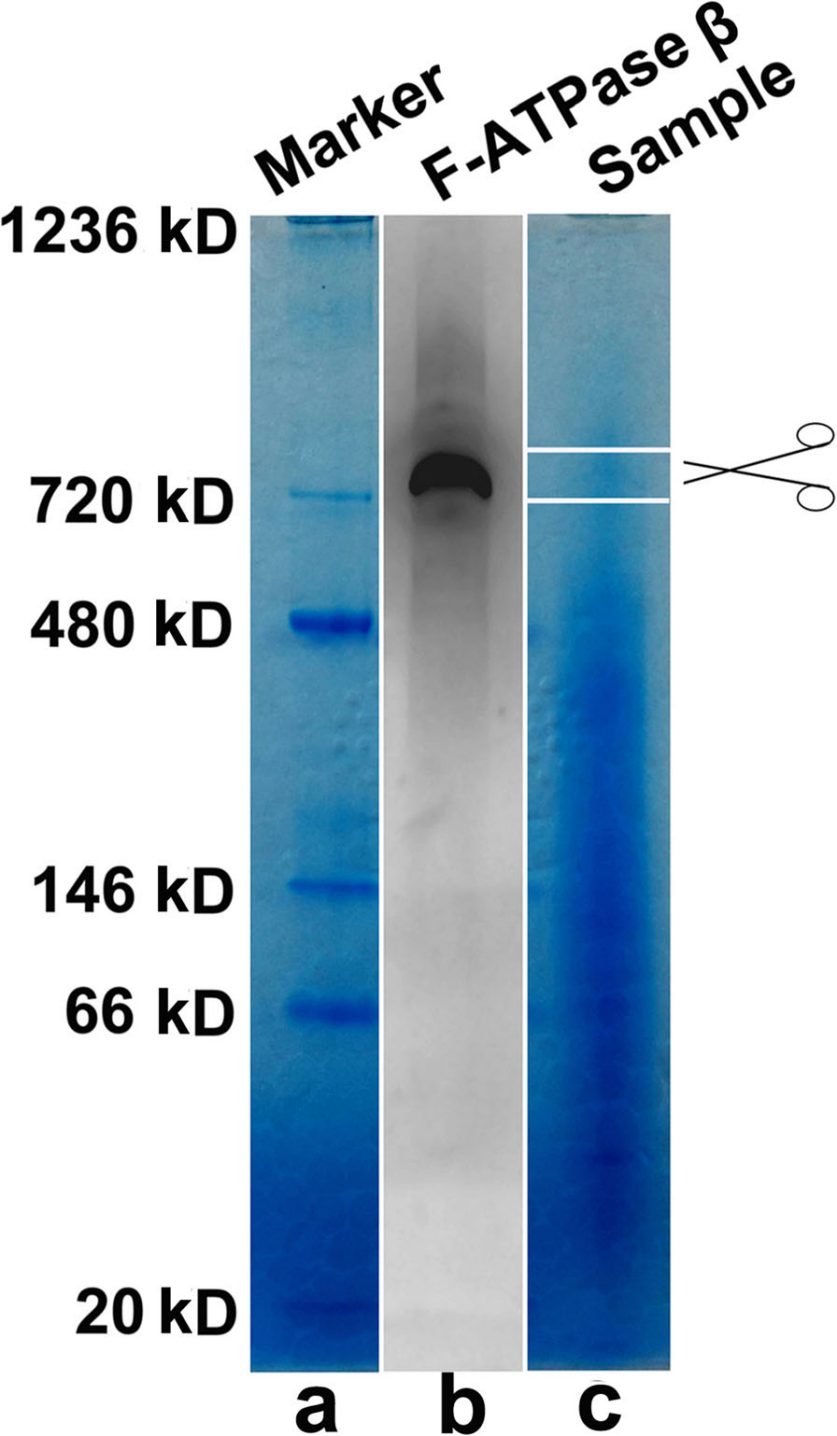
Analysis of F-ATPase-containing protein complexes in neutrophils. **a.** Representative protein marker in NativePAGE Novex 4–16% Bis-Tris gels. **b.** The F-ATPase-containing protein complexes in the gel were detected at approximately 750 kDa via immunoblotting with an F-ATPase F1 β-subunit antibody. **c**. The bands of interest were cut out according to their relative positions in a corresponding immunoblot and analyzed via mass spectrometry

After the purification and viability determination of neutrophils (Additional file 2), plasma membranes were purified and lysed in 2% digitonin to obtain protein complexes, and BN-PAGE was used to identify proteins that could interact with F-ATPase. Standard protein markers were separated on a 4–16% BN-PAGE gel (Fig. 1a). To determine the localization of the F-ATPase-containing protein complex in the BN-PAGE gel, the proteins in the gel were transferred to a PVDF membrane and detected by western blot analysis with an antibody against the F-ATPase F1 β-subunit. The molecular weight of the identified F-ATPase-containing protein complex was approximately 750 kDa (Fig. 1b), which was greater than that of the classical mitochondrial F-ATPase observed by BN-PAGE [26], suggesting that F-ATPase was part of a polyprotein complex. To further identify the components of this F-ATPase-containing protein complex, the F-ATPase-containing band was cut and analyzed with MS (Fig. 1c).

**Fig. 2:**
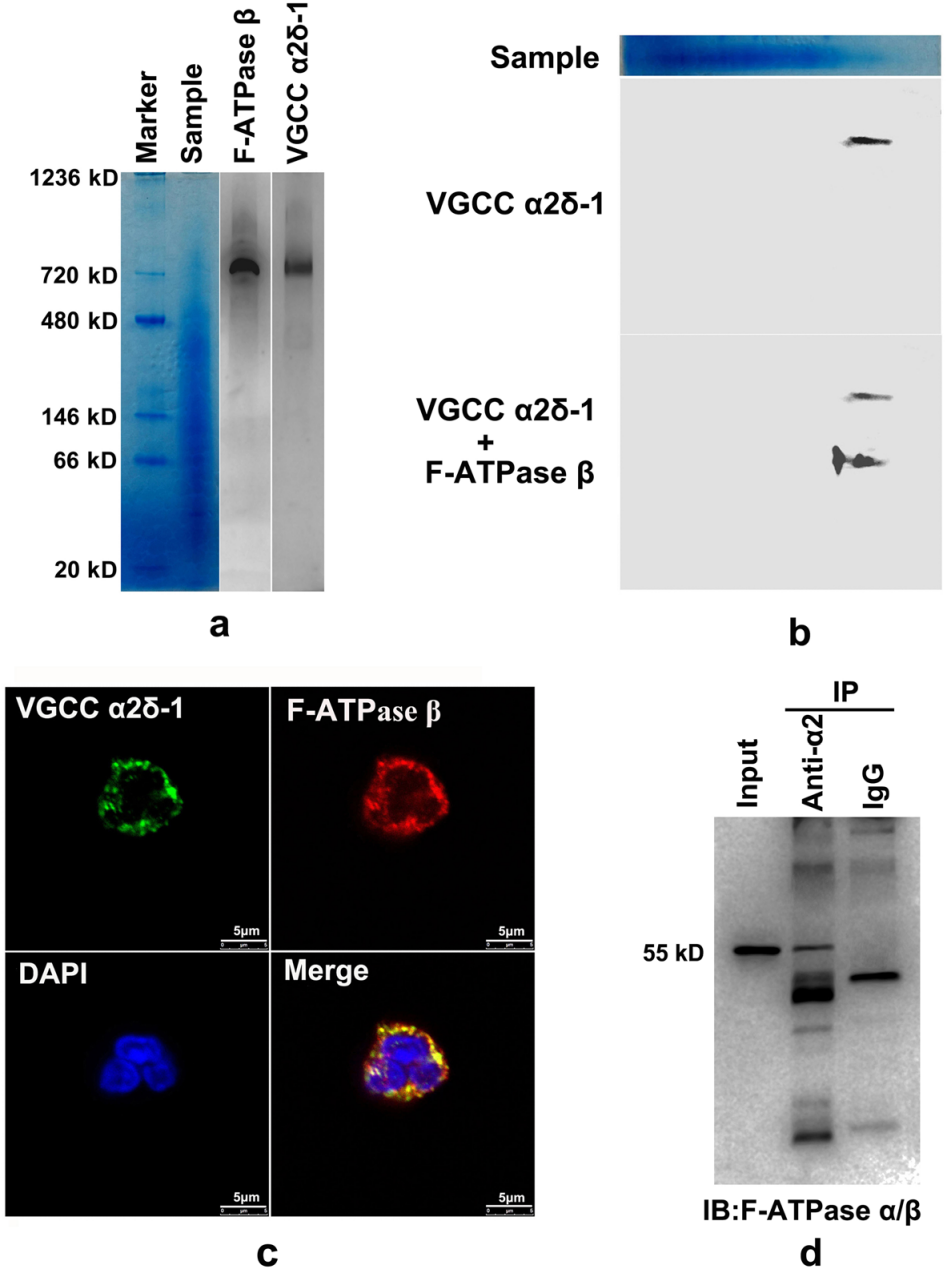
F-ATPase/VGCC protein complex verification. **a.** 1D immunoblot analysis of the VGCC α2δ-1 subunit in neutrophils. The VGCC α2δ-1 subunit-containing protein complexes in a NativePAGE Novex 4–16% Bis-Tris gel were detected at approximately 750 kDa via immunoblotting, the same position as the F-ATPase F1 β-subunit. **b.** 2D immunoblot analysis of the F-ATPase and VGCC protein complex. Plasma membrane lysed by 2% digitonin was loaded and run on a BN-PAGE gel. One lane (Sample) was cut and equilibrated in SDS loading buffer, followed by fixation on a SDS-PAGE gel for 2D electrophoretic analysis. Two separate immunoblot analyses with a primary antibody against the VGCC α2δ-1 subunit or the F-ATPase F1 β-subunit were performed. The F-ATPase F1 β-subunit appears vertically below the VGCC α2δ-1 subunit in the same PVDF membrane. **c.** Immunofluorescence colocalization analysis of F-ATPase and VGCC. Representative images show that the VGCC α2δ-1 subunit and F-ATPase F1 β-subunit colocalized on the plasma membrane of neutrophils. **d.** Immunoprecipitation of VGCC α2δ-1 subunit and F-ATPase from plasma membrane protein in human neutrophils. Samples were immunoprecipitated with a mouse monoclonal antibody against α2 of the VGCC α2δ-1 subunit. F-ATPase was detected with a mouse monoclonal antibody against the F-ATPase F1 α-subunit or β-subunit and an HRP-conjugated goat anti-mouse IgG light chain specifically bound antibody. IgG control experiment did not detect F-ATPase

After MS and bioinformatic analysis, VGCC α2δ-1 (UniProt accession number: P54289) was identified as a binding partner of F-ATPase. The identified peptide information of VGCC α2δ-1 is shown in Table 1. The complete protein amino acid sequence information and the MS/MS spectrum of the highest scored unique peptide (HLVNISVYAFNK) are shown in Additional file 3. To further confirm that F-ATPase and VGCC can form a protein complex, we performed 1D and 2D immunoblot verification. For 1D immunoblot analysis, protein complexes on the BN-PAGE gel were directly transferred to a PVDF membrane, and antibodies against the VGCC α2δ-1 subunit were applied. As shown in Fig. 2a, the VGCC α2δ-1 subunit appeared at the same site as the F-ATPase F1 β-subunit in two separate lanes of the BN-PAGE gel. For 2D immunoblot analysis, the BN-PAGE gel was cut out and equilibrated in SDS loading buffer to dissociate the protein complexes, followed by a second dimension of SDS-PAGE separation and two separate immunoblot analyses. The recognition site of antibodies against the F-ATPase F1 β-subunit appears vertically below the recognition site of antibodies against VGCC α2δ-1 in a single PVDF membrane (Fig. 2b). In addition, immunofluorescence analysis showed that the α2δ-1 subunit and the F-ATPase β-subunit colocalize on the cell surface rather than in intracellular mitochondria (Fig. 2c). To further verify whether the F-ATPase and VGCC α2δ-1 are physically linked, immunoprecipitation analysis with a mouse monoclonal antibody against α2 of the VGCC α2δ-1 subunit and nano-HPLC MS/MS analysis were applied. As shown in Additional files 4 and 5, F-ATPase F1 α and β subunits were identified from the anti α2 antibody-bound magnetic beads, which were incubated in lysate of the neutrophil plasma membrane. In addition, the parallel magnetic bead-bound proteins were denatured in SDS-PAGE reducing buffer for immunoblotting verification with a monoclonal antibody against the F-ATPase F1 α and β subunits. As expected, the band of the F-ATPase F1 α or β subunit was found in the lane that immunoprecipitated with anti α2 antibody. A negative control in the presence of monoclonal mouse IgG did not detect the F-ATPase F1 α or β subunit (Fig. 2d). Taken together, these results suggest that F-ATPase and VGCC can form a protein complex on the plasma membrane of neutrophils.

**Table 1:** Peptides of VGCCA2D1 identified by BN-PAGE

Start-End	-10lgP	PPM	Mass	Peptide
258–269	20.72	−4.7	1260.6925	VDVSGSVSGLTLK
557–571	27.44	−1.1	1733.8723	VTLDFLDAELENDIK^*^
635–643	22.08	1.6	1060.5400	LEETITQAR^*^
885–896	32.82	10.5	1404.7401	HLVNISVYAFNK^*^

*PPM* parts per million. ^*^, unique peptide

### Ca_v_2.3 is the main isoform of VGCC expressed in human neutrophils

**Fig. 3:**
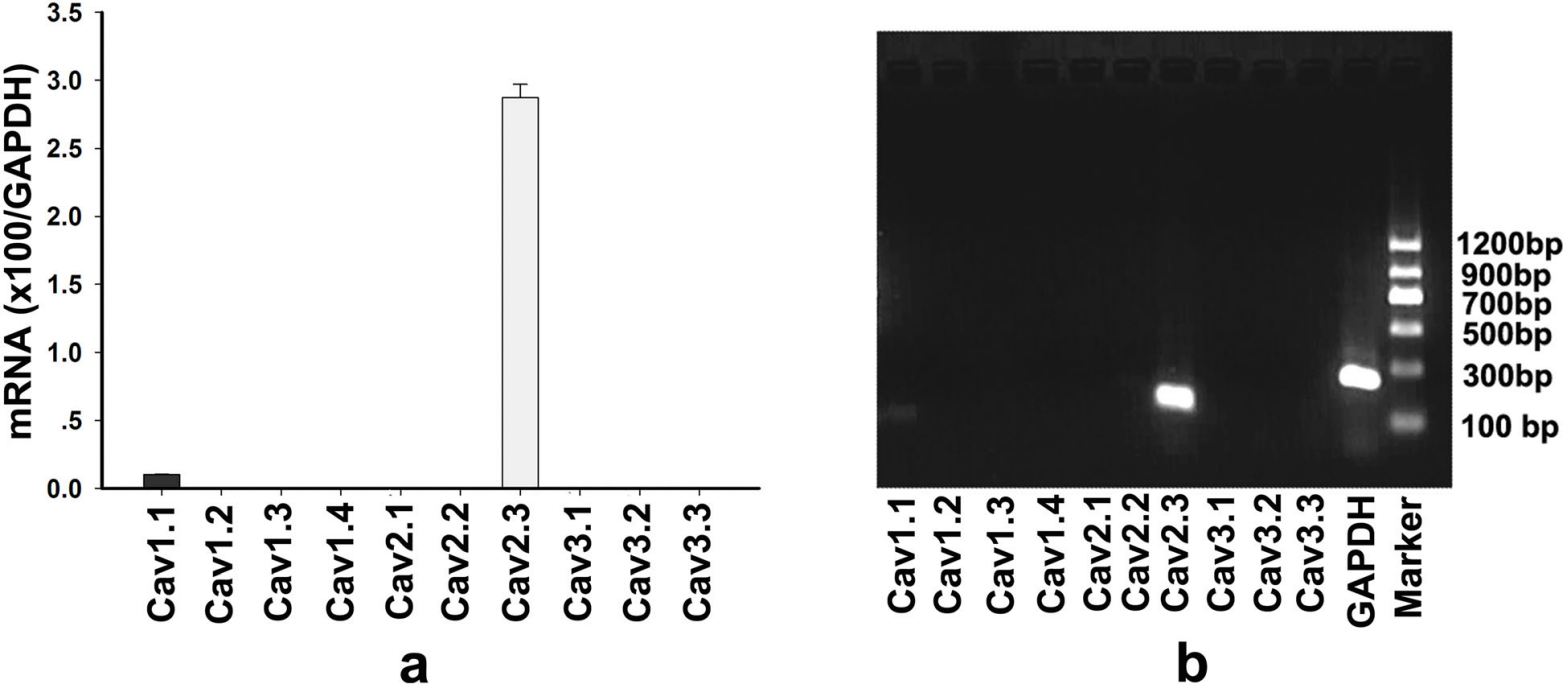
Expression of all 10 reported types of VGCCs in neutrophils. **a.** Quantitative real-time RT-PCR analysis of all 10 reported types of VGCCs. mRNA expression was determined relative to GAPDH in human neutrophils. The data are presented as the mean ± s.d. **b.** Agarose gel electrophoresis analysis. Samples were products of quantitative real-time RT-PCR without melt curve analysis

VGCC is composed of the α1, α2δ, β, and γ subunits. Ten types of VGCC have been reported with different α1 subunits [27]. The α2δ subunit is a key auxiliary subunit of VGCC. Previous studies have reported that α2δ-1, one of four isoforms of the α2δ subunit, can alter the function of various VGCCs as an auxiliary subunit [28–31]. Therefore, we next asked which kind of VGCC is expressed in neutrophils. All 10 known VGCCs were analyzed by real-time RT-PCR to assess the relative expression of the mRNAs of VGCCs in human neutrophils. The results indicated that Ca_v_2.3 (R-type VGCC)-derived mRNA was primarily expressed in neutrophils (Fig. 3a). These results were further confirmed by agarose gel electrophoresis (Fig. 3b).

### **Extracellular** Ca^2+^**influx is regulated by the F-ATPase/Ca**_**v**_**2.3**

**Fig. 4:**
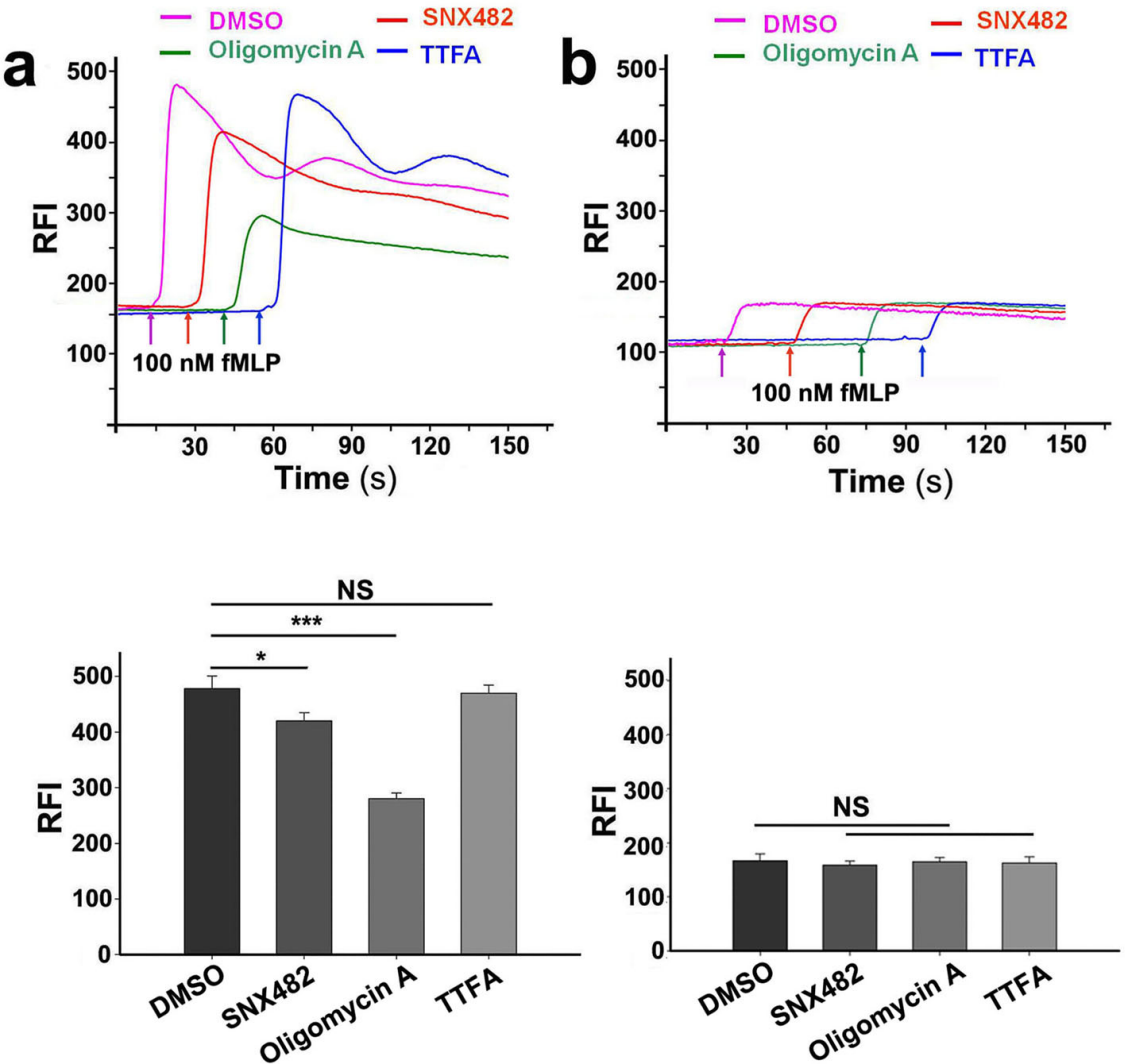
F-ATPase/Ca_v_2.3 complexes modulate extracellular Ca^2+^ influx. (**a**/left panel) Representative relative fluorescence intensity (RFI, Ex/Em = 488/525 nm) of Fluo-4-loaded neutrophils incubated in Ca^2+^-containing HBSS, with 0.1% DMSO used as a control, 400 nM SNX482 or 50 μg/ml oligomycin A used as an F-ATPase/Ca_v_2.3 complex inhibitor and 100 μM thenoyltrifluoroacetone (TTFA) used as a mitochondrial metabolic inhibitor (upper panel). The arrows indicate when 100 nM fMLP was applied. NS/*/*** - *P* > 0.05/< 0.05/< 0.001 versus the DMSO control (lower panel). The data are presented as the mean ± s.d. of similar triplicate determinations. (**b**/right panel) Representative RFI of Fluo-4-loaded neutrophils incubated in Ca^2+^-free HBSS with 0.1% DMSO, 400 nM SNX482, 50 μg/ml oligomycin A or 100 μM TTFA (upper panel). The arrows indicate when 100 nM fMLP was applied. NS - *P* > 0.05 versus the DMSO control (lower panel). The data are presented as the means ± s.d. of similar triplicate determinations

The above results indicate a probable physical association between F-ATPase and Ca_v_2.3 on the plasma membrane. We sought to further investigate the cell function of this interaction. The key role of VGCC is to regulate extracellular Ca^2+^ influx. Thus, we next asked whether F-ATPase can affect fMLP induced extracellular Ca^2+^ influx via the internal interaction with Ca_v_2.3. As shown in Fig. 4a (left panel), SNX482 (400 nM), an antagonist of Ca_v_2.3, significantly reduced (approximately 30%) the extracellular Ca^2+^ influx in Fluo-4-labeled neutrophils compared with that observed in the 0.1% DMSO control. Additionally, as expected, oligomycin A (50 μg/ml), an F-ATPase-specific inhibitor, also significantly decreased (approximately 75%) the extracellular Ca^2+^ influx in neutrophils. However, 100 μM thenoyltrifluoroacetone (TTFA), a mitochondrial metabolism inhibitor, did not significantly alter the extracellular Ca^2+^ influx. In addition, Fluo-4-loaded neutrophils in Ca^2+^-free HBSS were used to assess the intracellular Ca^2+^ release in different drug-induced cells (Fig. 4b/right panel). The figure shows that 400 nM SNX482, 100 mM TTFA and 50 μg/ml oligomycin A did not alter the intracellular Ca^2+^ release. Thus, these results suggest that the F-ATPase/Ca_v_2.3 complex is a functional interaction that can regulate extracellular Ca^2+^ influx.

### Neutrophil activation is regulated by the F-ATPase/Ca_v_2.3 functional complex

**Fig. 5:**
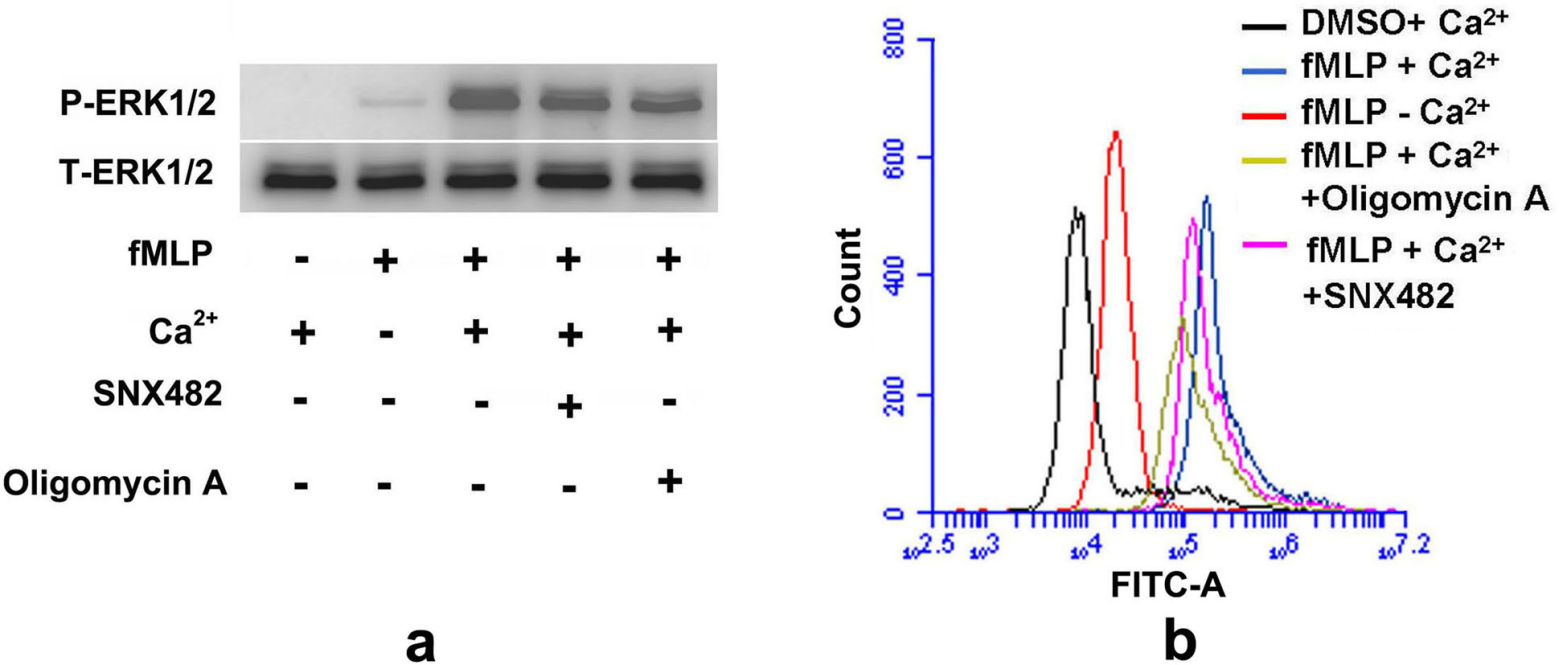
F-ATPase/Ca_v_2.3 functional complexes regulate ERK 1/2 phosphorylation and ROS production after fMLP activation of neutrophils. **a.** A representative western blot is presented to show the effect of F-ATPase/Ca_v_2.3 functional complexes on ERK 1/2 phosphorylation. Neutrophils were pretreated with 400 nM SNX482 and 50 μg/ml oligomycin A in Ca^2+^-containing HBSS for 30 min, followed by 1 min of 100 nM fMLP activation. Cells in Ca^2+^-containing HBSS or Ca^2+^-free HBSS incubated with or without fMLP were used as positive or negative controls, respectively. **b.** The effect of F-ATPase/Ca_v_2.3 functional complexes on ROS production was assessed by flow cytometry. DHR123-loaded neutrophils were pretreated with 400 nM SNX482 and 50 μg/ml oligomycin A in Ca^2+^-containing HBSS for 30 min, followed by 30 min of 100 nM fMLP incubation. Cells in Ca^2+^-containing HBSS or Ca^2+^-free HBSS incubated with or without fMLP were used as positive or negative controls, respectively

Calcium influx plays a key role in signal activation and various cell functions. Erk1/2 phosphorylation was considered one of the characteristics of activated neutrophils. Erk1/2 can be significantly phosphorylated within seconds in fMLP-activated neutrophils [32, 33]. In this work, as shown in Fig. 5a, the phosphorylation of ERK1/2 in fMLP-induced neutrophils was suppressed upon SNX482 and oligomycin A treatment in Ca^2+^-containing HBSS compared with fMLP-induced neutrophils in Ca^2+^-containing HBSS without inhibitors. However, Erk1/2 was not phosphorylated in Ca^2+^-containing HBSS without fMLP or slightly phosphorylated in Ca^2+^-free HBSS with fMLP. This result suggests that extracellular Ca^2+^ influx is essential for Erk1/2 phosphorylation. In addition, ROS production is another feature of activated neutrophils, which are involved in the antibacterial process [34]. We next asked whether ROS production was regulated by the F-ATPase/Ca_v_2.3 functional complex. DHR123, a fluorescent indicator of cytosolic ROS, was used to assess the effects of an F-ATPase/Ca_v_2.3 functional complex inhibitor on cytosolic ROS. As expected, quantitative flow cytometry analysis showed that the fMLP-induced activation of neutrophils caused the production of abundant cytosolic ROS in Ca^2+^-containing HBSS, whereas neutrophils incubated with SNX482 and oligomycin A exhibited reduced cytosolic ROS production (Fig. 5b). Cells incubated in Ca^2+^-containing HBSS without fMLP or in Ca^2+^-free HBSS with fMLP produced very few cytosolic ROS (Fig. 5b), suggesting that extracellular Ca^2+^ influx is important for cytosolic ROS production and that the F-ATPase/Ca_v_2.3 functional complex can modulate neutrophil activation by regulating Ca^2+^ influx.

### F-ATPase regulates neutrophil accumulation of LPS-induced acute lung injury in mice

**Fig. 6:**
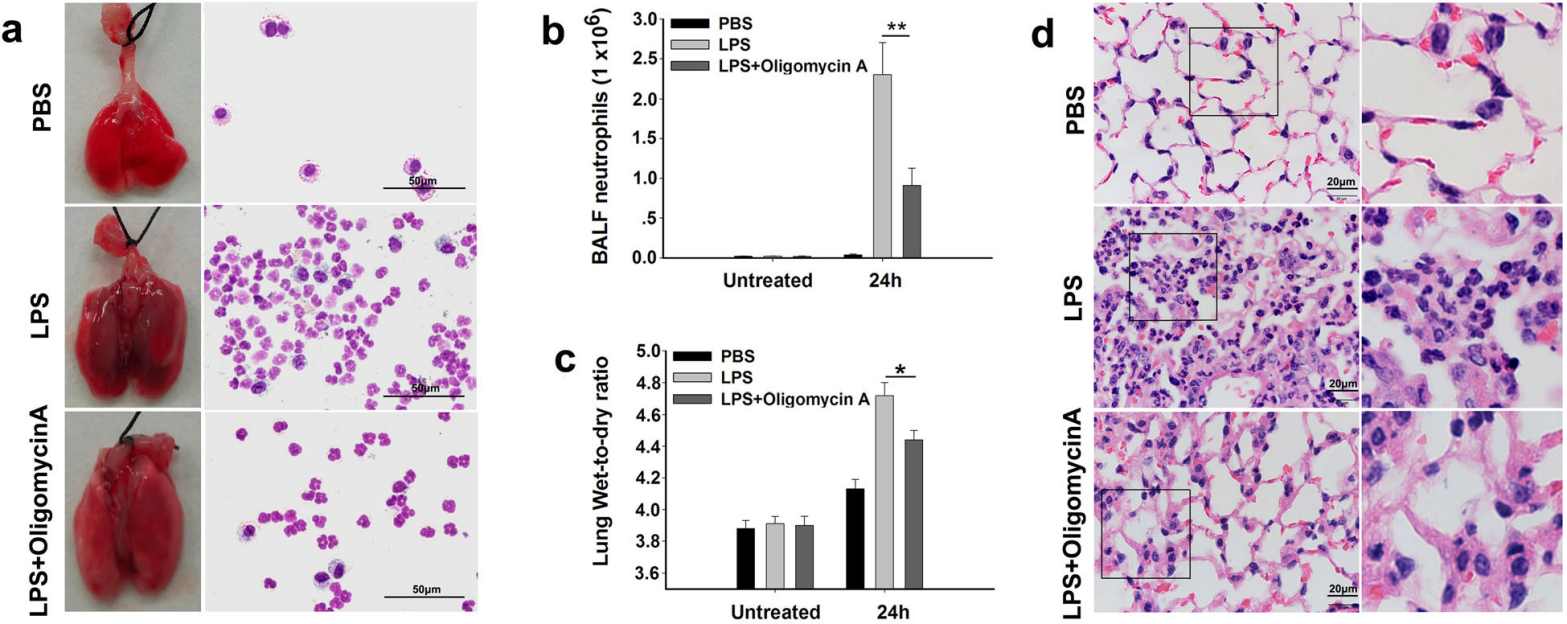
Inhibition of F-ATPase reduces neutrophil accumulation and reduces lung injury in LPS-induced lung inflammation. Mice were intratracheally instilled with LPS (5 mg/kg body weight) and sacrificed after 24 h. **a.** Representative images of the lungs of mice from different treatments and corresponding total cells in BALF, which were stained with a Wright-Giemsa stain using cytospin preparations. **b.** The total number of pulmonary neutrophils in BALF. **P < 0.01 versus the LPS mice. The data are presented as the mean ± s.d (*n* = 5). **c.** The lung wet-to-dry ratio was measured 24 h after LPS instillation. *P < 0.05 versus the LPS mice. The data are presented as the mean ± s.d (*n* = 5). **d.** Representative hematoxylin and eosin (H&E)-stained images of lung tissues and a corresponding 2x enlarged grid show emigrated neutrophils in the pulmonary parenchyma

Inflammation-induced tissue damage is an undesirable result of immune defenses. During the acute phase of lung infection, neutrophil infiltration and accumulation are key causes of lung injury [35]. To investigate the role of F-ATPase in neutrophil accumulation during pulmonary damage, an LPS-induced lung injury mouse model was developed by intratracheal instillation of LPS. As shown in Fig. 6a, the lungs of LPS-challenged mice exhibited inflammatory-like morphological changes compared with mice treated with PBS. Subsequently, the number of neutrophils in BALF samples was assessed. During lung injury, the number of neutrophils in BALF reached more than 2 × 10^6^ cells per lung 24 h after LPS instillation (Fig. 6b). However, LPS-induced neutrophil accumulation was significantly reduced in the oligomycin A-treated mice (Fig. 6b). In addition, the lung wet-to-dry ratio, which measures the change in capillary permeability, was reduced in the oligomycin A-treated mice compared to those treated with LPS alone (Fig. 6c). Furthermore, the emigrated neutrophils in alveolar spaces were also assessed by morphometry of lung tissue sections through H&E staining. Very few neutrophils were observed in the alveolar air spaces of PBS-treated mice, whereas LPS induced substantial neutrophil accumulation in alveolar air spaces (Fig. 6d). In addition, oligomycin A treatment significantly decreased the number of neutrophils in the alveolar air spaces in LPS-treated mice (Fig. 6d). Taken together, these results reveal that inhibition of F-ATPase by oligomycin A protects hosts from LPS-induced lung damage caused by neutrophil accumulation.

## Discussion

In the present study, the interaction between F-ATPase and the VGCC α2δ-1 subunit in the plasma membrane of neutrophils was demonstrated. The α2δ subunits are important auxiliary subunits of several kinds of VGCCs [36]. These subunits can increase the VGCC density on the plasma membrane and regulate Ca^2+^ currents [30, 37]. Previous reports showed that α2δ-1, an isoform of the α2δ subunit, can interact with low-density lipoprotein receptor-related protein 1 [38], the BK channel [39], thrombospondins [40] and N-methyl-d-aspartate receptors [41] in different cells and with different domains. Uriarte et al. reported the expression of the α2δ-1 subunit in neutrophils for the first time in a proteomic database without further verification when they studied the proteome of secretory vesicle membranes and plasma membranes of human neutrophils [42]. In this work, in addition to verifying the presence of α2δ-1 on the neutrophil surface, our results also indicated that there may exist a physical connection between F-ATPase and VGCC α2δ-1 via the domain interaction of the F1 part (α and β subunits) and the α2 part. In other words, F-ATPase is likely to regulate the function of VGCC α2δ-1 through conformational changes because during the process of ATP generation, the conformation of the F1 part is constantly changing.

Ten kinds of VGCCs have been reported in different cell types; however, the types of VGCCs expressed by neutrophils are still unknown. Real-time RT-PCR analysis of all 10 reported types of VGCCs confirmed that Ca_v_2.3 (R-type VGCC) is the primary type of VGCC expressed in neutrophils. These results suggest that F-ATPase may form a protein complex with Ca_v_2.3 via interactions with auxiliary α2δ-1 subunits. Ca_v_2.3 channels are high-threshold voltage-gated channels, and the activation potential of Ca_v_2.3 is between − 25 and − 40 mV [43]. Therefore, we next asked whether the change in membrane potential caused by fMLP-induced activation of neutrophils is sufficient to induce the activation of Ca_v_2.3. Seligmann et al. measured neutrophil membrane potential by using two indirect probes of membrane potential and found that the average resting membrane potential of human neutrophils is − 45 ± 2 mV [44]. They also reported that neutrophils were activated by fMLP in the range from 1 nM to 1 mM peptide, with 100 nM fMLP producing a maximal response [44]. After being activated by 100 nM fMLP, normal human neutrophils have a remarkable degree of depolarization, and the peak membrane potential can reach approximately − 20 mV [44]. This level of membrane potential is sufficient to activate Ca_v_2.3. Our further data showed that this functional complex participates in the regulation of extracellular Ca^2+^ influx and modulation of Erk1/2 phosphorylation and ROS production in fMLP-activated neutrophils. In other words, the F-ATPase/Ca_v_2.3 complex can regulate further neutrophil activation.

The F-ATPase/Ca_v_2.3 complex regulates Ca^2+^ influx, making it easier to understand the mechanism by which F-ATPase modulates cytosolic pH. In previous studies, we reported that F-ATPase can regulate cytosolic pH in fMLP-activated neutrophils [20]. However, earlier studies have confirmed that Na^+^/H^+^ exchanger 1 (NHE1) plays a key role in the regulation of cytosolic protons in activated neutrophils [45, 46]. How NHE1 participates in the regulation of cytosolic pH initiated by F-ATPase is still unclear. The results of this study indicate that a probable explanation for this phenomenon is that F-ATPase regulates the activation of NHE1 by modulating the influx of Ca^2+^, thereby regulating cytosolic pH because NHE1 activation requires Ca^2+^ influx via either the protein kinase C pathway [47, 48] or the calmodulin pathway [49, 50].

Another remaining question is why the maximum inhibitory effect of SNX482 (400 nm) on Ca^2+^ influx was significantly lower than that of oligomycin A (50 μg/ml) in the present experimental system. This result suggests that F-ATPase may inhibit Ca^2+^ influx through another pathway in addition to its interaction with Ca_v_2.3. Our previous studies showed that F-ATPase can regulate extracellular ATP concentrations [20]. Additionally, several kinds of P2 receptors, which are ATP-gated nonselective cation channels, were found on the surface of neutrophils [51], and various reports have shown that P2 receptors are involved in extracellular Ca^2+^ influx [52, 53]. Therefore, it is tempting to hypothesize that neutrophil-surface F-ATPase can indirectly regulate extracellular Ca^2+^ influx through the P2 receptor by modulating the extracellular ATP concentration. Two key problems must be resolved if we investigate the F-ATPase–ATP–P2 receptor–Ca^2+^ influx axis in neutrophils. (i) Only ATP concentrations in the extracellular medium were measured in previous studies on extracellular ATP production by neutrophils [51, 54, 55]. This method cannot accurately reflect real-time ATP concentrations at the cell surface because some of the produced ATP is not released into the extracellular medium. (ii) Ecto-nucleoside triphosphate diphosphohydrolase 1 (CD39) exists on the surface of neutrophils [56]. The effects of CD39 on cell surface ATP production need to be eliminated or controlled. Thus, further research on the F-ATPase–ATP–P2 receptor–Ca^2+^ influx pathway on the surface of neutrophils requires a cleverer design.

**Fig. 7:**
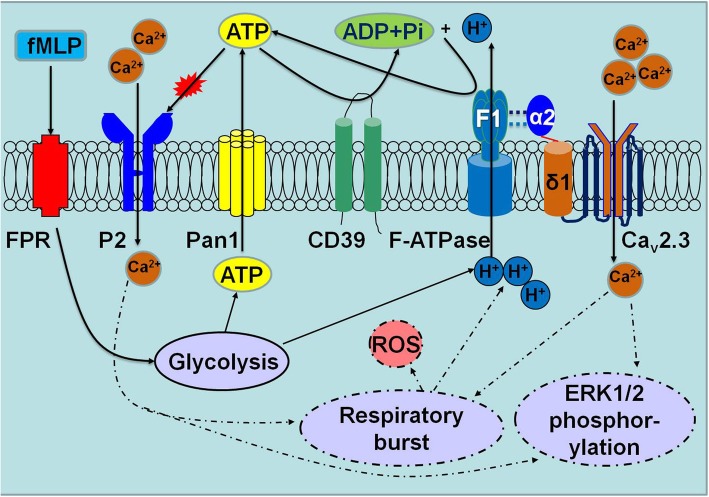
Proposed mechanism of cell surface F-ATPase-mediated regulation of Ca^2+^ influx. Stimulation with FPR triggers glycolysis to generate bulk intracellular protons and ATP that can be exported via F-ATPase, the pan1 channel, etc. Subsequently, ATP is hydrolyzed by CD39 to generate ADP and inorganic phosphate, which can be used to regenerate extracellular ATP with intracellular protons exported by F-ATPase to prolong cell surface ATP signaling and promote intracellular alkalization. The produced ATP indirectly regulates the P2 receptor to regulate Ca^2+^ influx. In addition, F-ATPase itself will affect Ca^2+^ influx by interacting with Ca_v_2.3. Calcium influx plays a key role in ERK1/2 phosphorylation and ROS production

Based on the results of this work and those of previous reports, we propose a role for plasma membrane F-ATPase in neutrophil Ca^2+^ influx regulation, as shown in Fig. 7. As previously reported, stimulation of chemoattractant receptor (FPR) on neutrophils with fMLP will induce increased glycolysis, and protons will be continually produced [46]. In addition, ATP will immediately be released through pannexin 1 channels (Pan1) at the leading edge and hydrolyzed to ADP and inorganic phosphate by CD39 [57–59]. Our previous study showed that cell surface F-ATPase translocated to the leading edge of fMLP-induced neutrophils and used extracellular ADP and inorganic phosphate for extracellular ATP regeneration. This process promotes an alkaline cytoplasm to facilitate cell migration and prolong the ATP signal at the cell surface [20]. The current work showed that the Ca^2+^ influx was altered by F-ATPase combined with Ca_v_2.3 via interaction through the α2δ-1 subunit of the latter. Furthermore, the regenerated ATP may act as a feedback signal to regulate Ca^2+^ influx via P2 receptors. Such regulation of Ca^2+^ influx by F-ATPase appears to affect the activation process of neutrophils, as Erk1/2 phosphorylation and the production of intracellular ROS were altered.

Neutrophils are an important component of the inflammatory response that characterizes acute lung injury [60]. The recruited and activated neutrophils in the lungs produce substantial ROS, which directly leads to oxygen stress [61]. In addition, neutrophil extracellular traps, which are formed during the late stages of neutrophil activation and are composed of granular proteins and decondensed chromatin that function to trap extracellular pathogens, can trap platelets and form thrombi in the lung microcirculatory system [62, 63]. Therefore, the more neutrophils that accumulate in the injured lung, the more secondary damage occurs to the lungs. In this study, inhibition of F-ATPase activation by oligomycin A significantly decreased neutrophil accumulation in BALF and alveolar air spaces. This inhibition, regardless of the morphology of the whole lung or the wet-to-dry ratio of the lung, has a protective effect on the LPS-challenged lungs of mice. This discovery may lead to new targeted therapeutic approaches against neutrophil-surface F-ATPase that balance pathogen clearance with unwanted inflammation-induced tissue damage.

## Conclusions

In summary, although details of the regulatory mechanism underlying cell surface F-ATPase control are unknown, the results of the present study indicated that surface F-ATPase of neutrophils may be involved in the regulation of extracellular Ca^2+^ influx and neutrophil activation by interacting with Ca_v_2.3. These observations of the positive impacts of F-ATPase inhibition in the LPS-induced pulmonary damage model point to the potential possibility of its use as a therapeutic target for neutrophil-related disease.

## Supplementary information

**Additional file 1.** Real-Time RT-PCR Primers obtained from PrimerBank (https://pga.mgh.harvard.edu/primerbank).

**Additional file 2.** Purification and viability determination of human peripheral blood neutrophils. **a.** A representative image of purified neutrophils with Wright-Giemsa staining. The average purity of the cells exceeded 95% under multiple microscopic views. The black arrow may indicate a contaminating lymphocyte. **b.** A representative picture of purified neutrophils from a trypan blue exclusion assay. The average viability of the purified cells was > 98%, as assessed in several microscopic views. The black arrows show blue and swollen dead neutrophils.

**Additional file 3.** The complete protein amino acid sequence and MS/MS spectrum of the highest scored unique peptide in Table 1.

**Additional file 4. **LC-MS-identified peptides of F-ATPase α and β subunits from protein complexes obtained via immunoprecipitation with an antibody against α2 of the VGCC α2δ-1 subunit. PPM, parts per million. *, unique peptide.

**Additional file 5.** The complete protein amino acid sequence and MS/MS spectrum of the highest scored unique peptide of F-ATPase α and β subunits in Additional file 4.

## Data Availability

The datasets used and/or analyzed during the current study are available from the corresponding author on reasonable request.

## Abbreviations

BAL: Bronchoalveolar lavage
BALF: Bronchoalveolar lavage fluid
BN-PAGE: Blue native polyacrylamide
F-ATPase: F0F1 ATP synthase
FDR: False discovery rate
FSC: Forward light scatter
LPS: Lipopolysaccharide
MS: Mass spectrometry
NHE1: Na^+^/H^+^ exchanger 1
RFI: Relative fluorescence intensity
ROS: Reactive oxygen species
SSC: Side light scatter
VGCC: Voltage-gated calcium channel

## References

[1] Fossati G, Moulding DA, Spiller DG, Moots RJ, White MR, Edwards SW (2003). The mitochondrial network of human neutrophils: role in chemotaxis, phagocytosis, respiratory burst activation, and commitment to apoptosis. J Immunol.

[2] Maianski NA, Geissler J, Srinivasula SM, Alnemri ES, Roos D, Kuijpers TW (2004). Functional characterization of mitochondria in neutrophils: a role restricted to apoptosis. Cell Death Differ.

[3] Borregaard N, Herlin T (1982). Energy metabolism of human neutrophils during phagocytosis. J Clin Invest.

[4] Edwards SW, Hallett MB, Campbell AK (1984). Oxygen-radical production during inflammation may be limited by oxygen concentration. Biochem J.

[5] Rodríguez-Espinosa O, Rojas-Espinosa O, Moreno-Altamirano MM, López-Villegas EO, Sánchez-García FJ (2015). Metabolic requirements for neutrophil extracellular traps formation. Immunology.

[6] Bao Y, Ledderose C, Seier T, Graf AF, Brix B, Chong E, Junger WG (2014). Mitochondria regulate neutrophil activation by generating ATP for autocrine purinergic signaling. J Biol Chem.

[7] Okuno D, Iino R, Noji H (2011). Rotation and structure of FoF1-ATP synthase. J Biochem.

[8] Junge W, Nelson N (2015). ATP synthase. Annu Rev Biochem.

[9] Perry SW, Norman JP, Barbieri J, Brown EB, Gelbard HA (2011). Mitochondrial membrane potential probes and the proton gradient: a practical usage guide. Biotechniques.

[10] Jethwaney D, Islam MR, Leidal KG, Bernabe BVD, Campbell KP, Nauseef WM, Gibson BW (2007). Proteomic analysis of plasma membrane and secretory vesicles from human neutrophils. Proteome Sci.

[11] Uriarte SM, Powell DW, Luerman GC, Merchant ML, Cummins TD, Jog NR, Ward RA, Mcleish KR (1808). Comparison of proteins expressed on secretory vesicle membranes and plasma membranes of human neutrophils. J Immunol.

[12] Chi SL, Pizzo SV (2006). Cell surface F1Fo ATP synthase: a new paradigm?. Ann Med.

[13] Kim BW, Choo HJ, Lee JW, Kim JH, Ko YG (2004). Extracellular ATP is generated by ATP synthase complex in adipocyte lipid rafts. Exp Mol Med.

[14] Martinez LO, Jacquet S, Esteve J-P, Rolland C, Cabezón E, Champagne E (2003). Ectopic β-chain of ATP synthase is an apolipoprotein A-I receptor in hepatic HDL endocytosis. Nature.

[15] Moser TL, Kenan DJ, Ashley TA, Roy JA, Goodman MD, Misra UK, Cheek DJ, Pizzo SV (2001). Endothelial cell surface F1-F0 ATP synthase is active in ATP synthesis and is inhibited by angiostatin. Proc Natl Acad Sci U S A.

[16] Moser TL, Stack MS, Asplin I, Enghild JJ, Hojrup P, Everitt L, Hubchak S, Schnaper HW, Pizzo SV (1999). Angiostatin binds ATP synthase on the surface of human endothelial cells. Proc Natl Acad Sci U S A.

[17] Xing SL, Yan J, Yu Z-H, Zhu C-Q (2010). Neuronal cell surface ATP synthase mediates synthesis of extracellular ATP and regulation of intracellular pH. Cell Biol Int.

[18] Benelli R, Morini M, Carrozzino F, Ferrari N, Minghelli S, Santi L, Cassatella M, Noonan DM, Albini A (2002). Neutrophils as a key cellular target for angiostatin: implications for regulation of angiogenesis and inflammation. FASEB J.

[19] Gao J, Li J, Feng C, Hu Z, Liu W, Liang S, Yin D (2013). Isolation technique and proteomic analysis of the erythrocyte ghosts of red-eared turtle (Trachemys scripta). Electrophoresis.

[20] Gao J, Zhang T, Kang Z, Ting W, Xu L, Yin D (2017). The F0F1 ATP synthase regulates human neutrophil migration through cytoplasmic proton extrusion coupled with ATP generation. Mol Immunol.

[21] Ilka W, Hans-Peter B, Hermann SG (2006). Blue native PAGE. Nat Protoc.

[22] Jia C, Haiyang T, Nissim H, Jingsong X, Ye RD (2010). Akt isoforms differentially regulate neutrophil functions. Blood.

[23] Wisniewski JR, Zougman A, Nagaraj N, Mann M (2009). Universal sample preparation method for proteome analysis. Nat Methods.

[24] van Pelt LJ, Van ZR, Weening RS, Roos D, Verhoeven AJ, Bolscher BG (1912). Limitations on the use of dihydrorhodamine 123 for flow cytometric analysis of the neutrophil respiratory burst. J Immunol Methods.

[25] Mei Shirley H. J, McCarter Sarah D, Deng Yupu, Parker Colleen H, Liles W. Conrad, Stewart Duncan J (2007). Prevention of LPS-Induced Acute Lung Injury in Mice by Mesenchymal Stem Cells Overexpressing Angiopoietin 1. PLoS Medicine.

[26] Klement P, Nijtmans LGJ, Vandenbogert C, Houstek J (1995). Analysis of oxidative phosphorylation complexes in cultured human fibroblasts and Amniocytes by blue-native-electrophoresis using Mitoplasts isolated with the help of Digitonin. Anal Biochem.

[27] Perezreyes E (2003). Molecular physiology of low-voltage-activated T-type calcium channels. Physiol Rev.

[28] Andrade A, Sandoval A, González-Ramírez R, Lipscombe D, Campbell KP, Felix R (2009). The alpha(2)delta subunit augments functional expression and modifies the pharmacology of Ca(V)1.3 L-type channels. Cell Calcium.

[29] Yasuda T, Chen L, Barr W, Mcrory JE, Lewis RJ, Adams DJ, Zamponi GW (2015). Auxiliary subunit regulation of high-voltage activated calcium channels expressed in mammalian cells. Eur J Neurosci.

[30] Cassidy JS, Ferron L, Kadurin I, Pratt WS, Dolphin AC (2014). Functional exofacially tagged N-type calcium channels elucidate the interaction with auxiliary α2δ-1 subunits. Proc Natl Acad Sci U S A.

[31] Baumann L, Gerstner A, Zong X, Biel M, Wahlschott C (2004). Functional characterization of the L-type Ca2+ channel Cav1.4α1 from mouse retina. Invest Ophthalmol Vis Sci.

[32] Liu X, Ma B, Malik AB, Tang H, Yang T, Sun B, Wang G, Minshall RD, Li Y, Zhao Y (2012). Bidirectional regulation of neutrophil migration by mitogen-activated protein kinases. Nat Immunol.

[33] Sandoval A, Triviños F, Sanhueza A, Carretta D, Hidalgo MA, Hancke JL, Burgos RA (2007). Propionate induces pH (i) changes through calcium flux, ERK1/2, p38, and PKC in bovine neutrophils. Vet Immunol Immunopathol.

[34] Rochael NC, Guimarães-Costa AB, Nascimento MT, Desouza-Vieira TS, Oliveira MP, Lf GES, Oliveira MF, Saraiva EM (2015). Classical ROS-dependent and early/rapid ROS-independent release of neutrophil extracellular traps triggered by Leishmania parasites. Sci Rep.

[35] Hou Qingming, Liu Fei, Chakraborty Anutosh, Jia Yonghui, Prasad Amit, Yu Hongbo, Zhao Li, Ye Keqiang, Snyder Solomon H., Xu Yuanfu, Luo Hongbo R. (2018). Inhibition of IP6K1 suppresses neutrophil-mediated pulmonary damage in bacterial pneumonia. Science Translational Medicine.

[36] Davies A, Hendrich J, Minh ATV, Wratten J, Douglas L, Dolphin AC (2007). Functional biology of the α2δ subunits of voltage-gated calcium channels. Trends Pharmacol Sci.

[37] Gurnett CA, Waard MD, Campbell KP (1996). Dual function of the voltage-dependent Ca 2+ channel α 2 δ subunit in current stimulation and subunit interaction. Neuron.

[38] Kadurin I, Rothwell SW, Lana B, NietoRostro M, Dolphin AC (2017). LRP1 influences trafficking of N-type calcium channels via interaction with the auxiliary α2δ-1 subunit. Sci Rep.

[39] Zhang FX, Gadotti VM, Souza IA, Chen L, Zamponi GW (2018). BK potassium channels suppress Cavα2δ subunit function to reduce inflammatory and neuropathic pain. Cell Rep.

[40] Eroglu Ç, Allen NJ, Susman MW, O'Rourke NA, Chan YP, Özkan E, Chakraborty C, Mulinyawe SB, Annis DS, Huberman AD (2009). Gabapentin receptor α2δ-1 is a neuronal Thrombospondin receptor responsible for excitatory CNS synaptogenesis. Cell.

[41] Chen J, Li L, Chen SR, Chen H, Xie JD, Sirrieh RE, Maclean DM, Zhang Y, Zhou MH, Jayaraman V (2018). The α2δ-1-NMDA receptor complex is critically involved in neuropathic pain development and gabapentin therapeutic actions. Cell Rep.

[42] Uriarte S, Powell D, Gc MM, Cummins T, Jog N, Ward R, Mcleish K (1808). Comparison of proteins expressed on secretory vesicle membranes and plasma membranes of human neutrophils. J Immunol.

[43] Gurkoff G, Shahlaie K, Lyeth B, Berman R (2013). Voltage-gated Calcium Channel antagonists and traumatic brain injury. Pharmaceuticals.

[44] Seligmann BE, Gallin JI (1980). Use of lipophilic probes of membrane potential to assess human neutrophil activation. Abnormality in chronic granulomatous disease. J Clin Invest.

[45] Simchowitz L (1985). Chemotactic factor-induced activation of Na+/H+ exchange in human neutrophils. II. Intracellular pH changes. J.biol.chem.

[46] Hisayoshi H, Orit A, R Todd A, Nicolas T, Wendy F, John O, Sergio G (2008). Na+/H+ exchange and pH regulation in the control of neutrophil chemokinesis and chemotaxis. Am J Physiol-Cell Physiol.

[47] Sardet C, Counillon L, Franchi A, Pouysségur J (1990). Growth factors induce phosphorylation of the Na+/H+ Antiporter, a glycoprotein of 110 kD. Science.

[48] Maly K, Strese K, Kampfer S, Ueberall F, Baier G, Ghaffari-Tabrizi N, Grunicke HH, Leitges M (2002). Critical role of protein kinase C α and calcium in growth factor induced activation of the Na + /H + exchanger NHE1. FEBS Lett.

[49] Wakabayashi S, Bertrand B, Shigekawa M, Fafournoux P, Pouysségur J (1994). Growth factor activation and "H(+)-sensing" of the Na+/H+ exchanger isoform 1 (NHE1). Evidence for an additional mechanism not requiring direct phosphorylation. J Biol Chem.

[50] Wakabayashi S, Bertrand B, Ikeda T, Pouysségur J, Shigekawa M (1994). Mutation of calmodulin-binding site renders the Na+/H+ exchanger (NHE1) highly H(+)-sensitive and Ca2+ regulation-defective. J Biol Chem.

[51] Yu C, Ross C, Yoshiaki I, Linda Y, Naoyuki H, Annelies Z, Victor N, Paul AI, Wolfgang GJ (2006). ATP release guides neutrophil chemotaxis via P2Y2 and A3 receptors. Science.

[52] Yamamoto K, Korenaga R, Kamiya A, Qi Z, Sokabe M, Ando J (2000). P2X(4) receptors mediate ATP-induced calcium influx in human vascular endothelial cells. Am J Physiol Heart Circ Physiol.

[53] Pankratov YV, Lalo UV, Krishtal OA (2002). Role for P2X receptors in long-term potentiation. J Neurosci.

[54] Yi B, Yu C, Carola L, Linglin L, Junger WG (2013). Pannexin 1 channels link chemoattractant receptor signaling to local excitation and global inhibition responses at the front and back of polarized neutrophils. J Biol Chem.

[55] Eltzschig HK, Macmanus CF, Colgan SP (2008). Neutrophils as sources of extracellular nucleotides: functional consequences at the vascular Interface. Trends Cardiovasc Med.

[56] Luca A, Pál P (2013). E Sylvester V, and Gy?Rgy H, CD39 and CD73 in immunity and inflammation. Trends Mol Med.

[57] Hayashi H, Aharonovitz O, Alexander RT, Touret N, Furuya W, Orlowski J, Grinstein S (2008). Na+/H+ exchange and pH regulation in the control of neutrophil chemokinesis and chemotaxis. Am J Phys Cell Phys.

[58] Chen Y, Corriden R, Inoue Y, Yip L, Hashiguchi N, Zinkernagel A, Nizet V, Insel PA, Junger WG (2006). ATP release guides neutrophil chemotaxis via P2Y2 and A3 receptors. Science.

[59] Corriden R, Chen Y, Inoue Y, Beldi G, Robson SC, Insel PA, Junger WG (2008). Ecto-nucleoside triphosphate Diphosphohydrolase 1 (E-NTPDase1/CD39) regulates neutrophil Chemotaxis by hydrolyzing released ATP to adenosine. J Biol Chem.

[60] Abraham E (2003). Neutrophils and acute lung injury. Crit Care Med.

[61] Min JH, Codipilly CN, Nasim S, Miller EJ, Ahmed MN (2012). Synergistic protection against hyperoxia-induced lung injury by neutrophils blockade and EC-SOD overexpression. Respir Res.

[62] Caudrillier A, Kessenbrock K, Gilliss BM, Nguyen JX, Marques MB, Monestier M, Toy P, Werb Z, Looney MR (2012). Platelets induce neutrophil extracellular traps in transfusion-related acute lung injury. J Clin Invest.

[63] Sil P, Hayes CP, Reaves BJ, Breen P, Quinn S, Sokolove J, Rada B (1981). P2Y6 receptor antagonist MRS2578 inhibits neutrophil activation and aggregated neutrophil extracellular trap formation induced by gout-associated monosodium Urate crystals. J Immunol.

