# Supplementary material for: F0F1 ATP synthase regulates extracellular calcium influx in human neutrophils by interacting with Cav2.3 and modulates neutrophil accumulation in the lipopolysaccharide-challenged lung
Source: Cell Commun Signal. 2020 Feb 4;18:19. doi: 10.1186/s12964-020-0515-3 (PMC7001235; doi:10.1186/s12964-020-0515-3)
Supplement: Supplementary file 1 — Additional file 1. Real-Time RT-PCR Primers obtained from PrimerBank (https://pga.mgh.harvard.edu/primerbank). [file 12964_2020_515_MOESM1_ESM.docx]

**Additional file 1,** Real-Time RT-PCR Primers obtained from PrimerBank (https://pga.mgh.harvard.edu/primerbank).

| **Receptor** | **PrimerBank ID** | | **Primer** | **Sequence (5’ to 3’)** |
| --- | --- | --- | --- | --- |
| CAV1.1 (L type) | | 110349766c1 | Forward | CTTCGAGACGATCATCTTGCTC |
|  | |  | Reverse | GCGGCTTCAATCGAGAAGACA |
| CAV1.2 (L type) | | 193788716c1 | Forward | GAAGCGGCAGCAATATGGGA |
|  | |  | Reverse | TTGGTGGCGTTGGAATCATCT |
| CAV1.3 (L type) | | 192807299c1 | Forward | CGCGAACGAGGCAAACTATG |
|  | |  | Reverse | TTGGAGCTATTCGGCTGAGAA |
| CAV1.4 (L type) | | 377823714c1 | Forward | GGAAGCCCTTCGACATCCTC |
|  | |  | Reverse | GTAGGCCACGATCTTGAGCAC |
| CAV2.1 (P/Q type) | | 148536843c1 | Forward | CGCTTCGGAGACGAGATGC |
|  | |  | Reverse | TGCGCCATTGACTGCTTGT |
| CAV2.2 (N type) | | 345091031c1 | Forward | GACAACGTCGTCCGCAAATAC |
|  | |  | Reverse | CCCGATGAAATAGGGCTCCG |
| CAV2.3 (R-type) | | 329663530c1 | Forward | CCATGTCCCGAAGACTGGAGA |
|  | |  | Reverse | CCATTGCGGAGGTAAGAGC |
| CAV3.1 (T type) | | 373838805c1 | Forward | TGTCTCCGCACGGTCTGTAA |
|  | |  | Reverse | AAGCCGGTTCCAAGTGTCTC |
| CAV3.2 (T type) | | 53832010c1 | Forward | ATGCTGGTAATCATGCTCAACTG |
|  | |  | Reverse | AAAAGGCGAAAATGAAGGCGT |
| CAV3.3 (T type) | | 51093858c1 | Forward | GGAGCTGATCCTCATGTCCC |
|  | |  | Reverse | CACGGGTTGCACACCATCT |
| GAPDH | | 378404907c1 | Forward | GGAGCGAGATCCCTCCAAAAT |
|  | |  | Reverse | GGCTGTTGTCATACTTCTCATGG |
