# Supplementary figures and images for: F0F1 ATP synthase regulates extracellular calcium influx in human neutrophils by interacting with Cav2.3 and modulates neutrophil accumulation in the lipopolysaccharide-challenged lung
Source: Cell Commun Signal. 2020 Feb 4;18:19. doi: 10.1186/s12964-020-0515-3 (PMC7001235; doi:10.1186/s12964-020-0515-3)

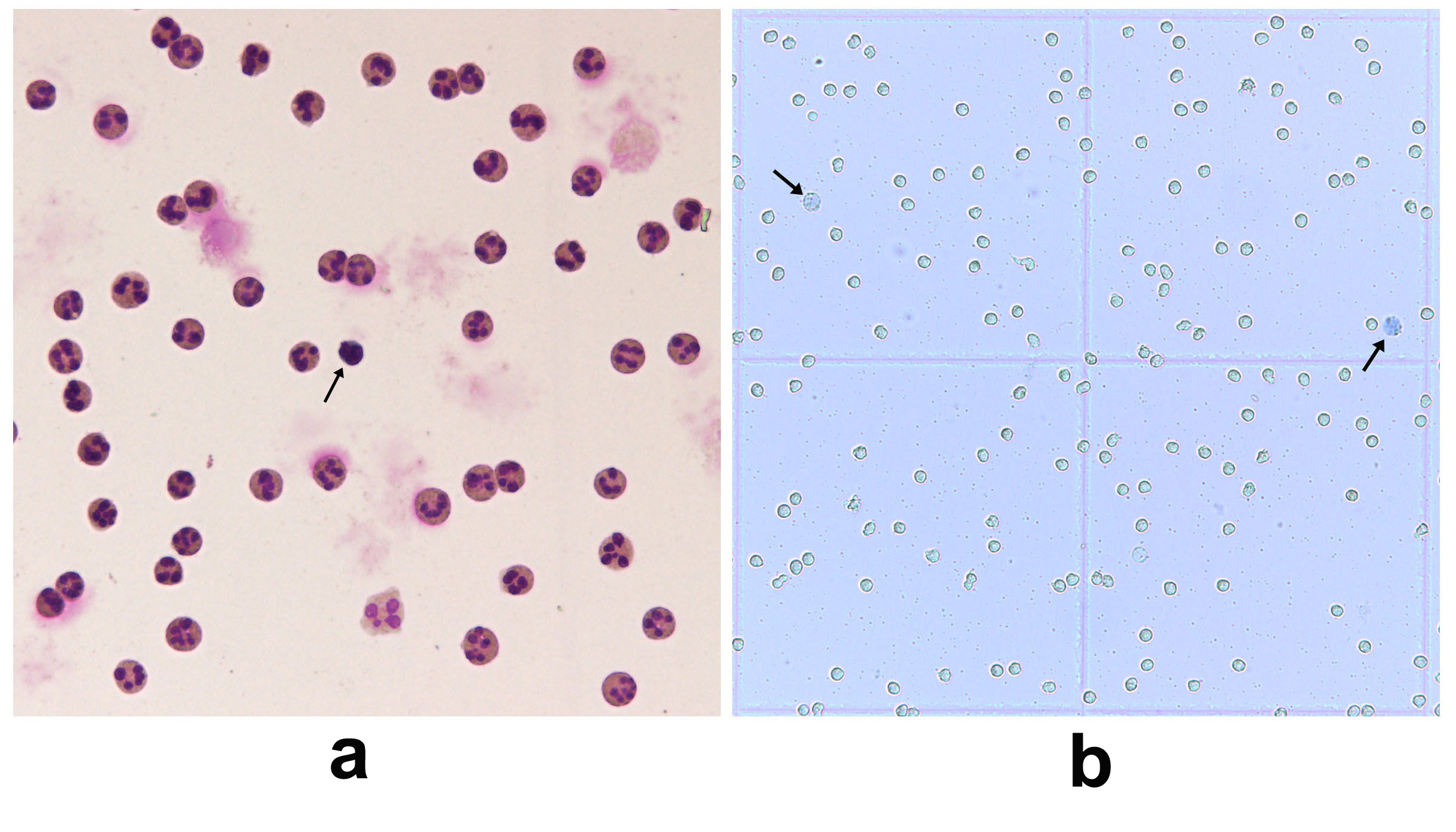

Supplement: Supplementary file 2 — Additional file 2. Purification and viability determination of human peripheral blood neutrophils. a. A representative image of purified neutrophils with Wright-Giemsa staining. The average purity of the cells exceeded 95% under multiple microscopic views. The black arrow may indicate a contaminating lymphocyte. b. A representative picture of purified neutrophils from a trypan blue exclusion assay. The average viability of the purified cells was > 98%, as assessed in several microscopic views. The black arrows show blue and swollen dead neutrophils. [file 12964_2020_515_MOESM2_ESM.jpg]

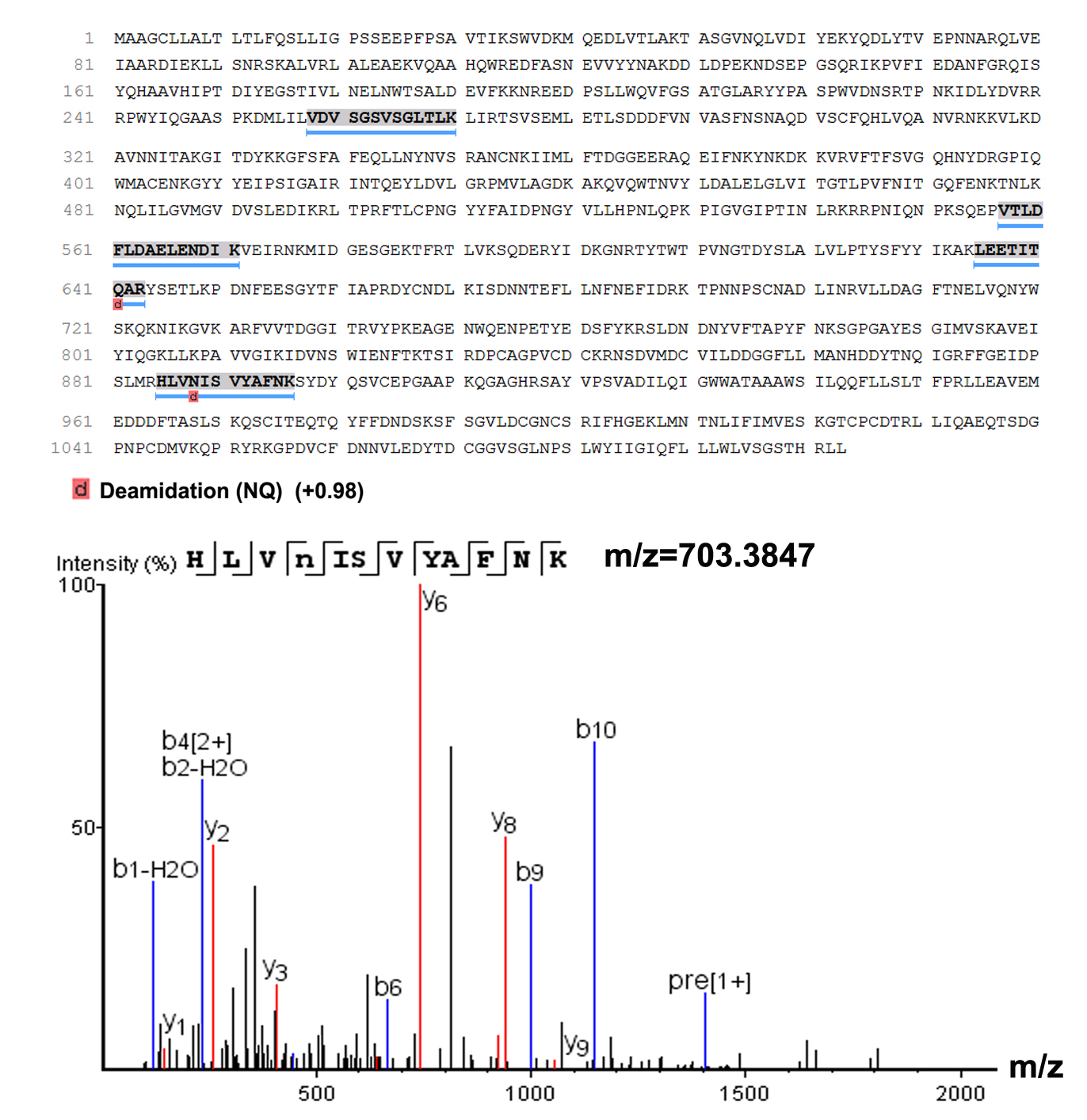

Supplement: Supplementary file 3 — Additional file 3. The complete protein amino acid sequence and MS/MS spectrum of the highest scored unique peptide in Table 1. [file 12964_2020_515_MOESM3_ESM.jpg]

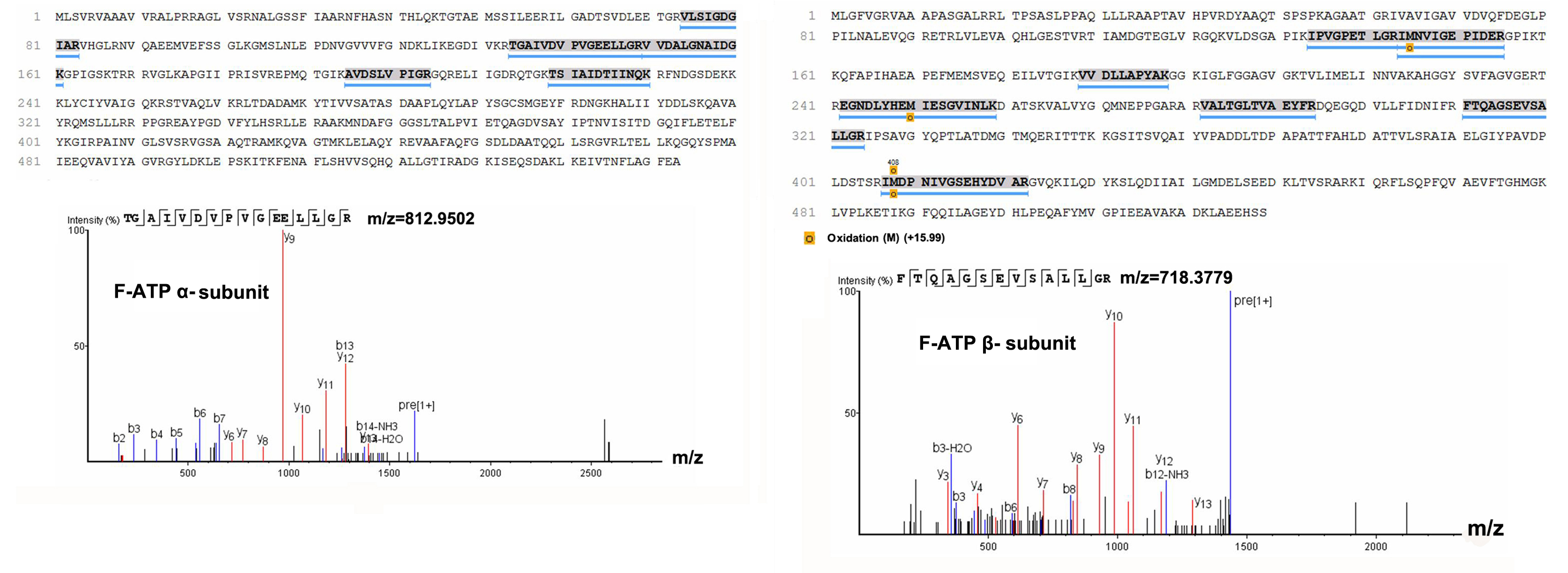

Supplement: Supplementary file 5 — Additional file 5. The complete protein amino acid sequence and MS/MS spectrum of the highest scored unique peptide of F-ATPase α and β subunits in Additional file 4. [file 12964_2020_515_MOESM5_ESM.jpg]
