# Supplementary material for: F0F1 ATP synthase regulates extracellular calcium influx in human neutrophils by interacting with Cav2.3 and modulates neutrophil accumulation in the lipopolysaccharide-challenged lung
Source: Cell Commun Signal. 2020 Feb 4;18:19. doi: 10.1186/s12964-020-0515-3 (PMC7001235; doi:10.1186/s12964-020-0515-3)
Supplement: Supplementary file 4 — Additional file 4. LC-MS-identified peptides of F-ATPase α and β subunits from protein complexes obtained via immunoprecipitation with an antibody against α2 of the VGCC α2δ-1 subunit. PPM, parts per million. *, unique peptide. [file 12964_2020_515_MOESM4_ESM.docx]

**Additional file 4.** LC-MS-identified peptides of F-ATPase α and β subunits from protein complexes obtained via immunoprecipitation with an antibody against α2 of the VGCC α2δ-1 subunit.

| F-ATPase α subunit | Start-End | -10lgP | PPM | Mass | Peptide |
| --- | --- | --- | --- | --- | --- |
|  | 134-149 | 61.68 | 1.6 | 1623.8832 | TGAIVDVPVGEELLGR^*^ |
|  | 150-161 | 53.76 | -2 | 1170.6244 | VVDALGNAIDGK^*^ |
|  | 195-204 | 50.33 | -0.4 | 1025.5869 | AVDSLVPIGR^*^ |
|  | 219-230 | 47.06 | 2.8 | 1315.7347 | TSIAIDTIINQK^*^ |
|  | 74-83 | 28.47 | 4 | 999.5713 | VLSIGDGIAR^*^ |
| F-ATPase β subunit | 311-324 | 58.36 | -3.8 | 1434.7467 | FTQAGSEVSALLGR^*^ |
|  | 189-198 | 52.42 | 0.7 | 1087.6277 | VVDLLAPYAK^*^ |
|  | 144-155 | 47.41 | 5 | 1400.6969 | IMNVIGEPIDER^*^ |
|  | 407-422 | 46.42 | 4.1 | 1830.8571 | IMDPNIVGSEHYDVAR^*^ |
|  | 282-294 | 46.31 | 7.4 | 1438.782 | VALTGLTVAEYFR^*^ |
|  | 134-143 | 31.93 | 1.2 | 1037.5869 | IPVGPETLGR^*^ |
|  | 242-259 | 19.72 | 3.6 | 2075.9834 | EGNDLYHEMIESGVINLK^*^ |

PPM, parts per million. *****, unique peptide
